# Removing the bottlenecks of cell culture metabolomics: fast normalization procedure, correlation of metabolites to cell number, and impact of the cell harvesting method

**DOI:** 10.1007/s11306-016-1104-8

**Published:** 2016-09-15

**Authors:** Caroline Muschet, Gabriele Möller, Cornelia Prehn, Martin Hrabě de Angelis, Jerzy Adamski, Janina Tokarz

**Affiliations:** 1 Helmholtz Zentrum München, German Research Center for Environmental Health, Institute of Experimental Genetics, Genome Analysis Center, Ingolstaedter Landstrasse 1, 85764 Neuherberg, Germany; 2 Lehrstuhl für Experimentelle Genetik, Technische Universität München, 85350 Freising-Weihenstephan, Germany; 3 German Center for Diabetes Research (DZD), 85764 Neuherberg, Germany

**Keywords:** Cell culture metabolomics, Normalization method, Harvesting, Metabolite–cell number correlation

## Abstract

**Introduction:**

Although cultured cells are nowadays regularly analyzed by metabolomics technologies, some issues in study setup and data processing are still not resolved to complete satisfaction: a suitable harvesting method for adherent cells, a fast and robust method for data normalization, and the proof that metabolite levels can be normalized to cell number.

**Objectives:**

We intended to develop a fast method for normalization of cell culture metabolomics samples, to analyze how metabolite levels correlate with cell numbers, and to elucidate the impact of the kind of harvesting on measured metabolite profiles.

**Methods:**

We cultured four different human cell lines and used them to develop a fluorescence-based method for DNA quantification. Further, we assessed the correlation between metabolite levels and cell numbers and focused on the impact of the harvesting method (scraping or trypsinization) on the metabolite profile.

**Results:**

We developed a fast, sensitive and robust fluorescence-based method for DNA quantification showing excellent linear correlation between fluorescence intensities and cell numbers for all cell lines. Furthermore, 82–97 % of the measured intracellular metabolites displayed linear correlation between metabolite concentrations and cell numbers. We observed differences in amino acids, biogenic amines, and lipid levels between trypsinized and scraped cells.

**Conclusion:**

We offer a fast, robust, and validated normalization method for cell culture metabolomics samples and demonstrate the eligibility of the normalization of metabolomics data to the cell number. We show a cell line and metabolite-specific impact of the harvesting method on metabolite concentrations.

**Electronic supplementary material:**

The online version of this article (doi:10.1007/s11306-016-1104-8) contains supplementary material, which is available to authorized users.

## Introduction

Metabolomics has long been used in studies related to human health, in which mostly body fluids were analyzed for various clinical indications (Cuperlović-Culf et al. 2010; Beckonert et al. 2007). However, it is increasingly applied to other matrices such as tissues (Römisch-Margl et al. 2012) and cells (e.g., immortalized cell lines, primary cells, or induced pluripotent stem cells) (Cuperlović-Culf et al. 2010; Kleinstreuer et al. 2011; Dettmer et al. 2011, 2013; Meissen et al. 2012; Berthon et al. 1993; Khoo and Al-Rubeai 2007; Ritter et al. 2008). Especially metabolomics with cultured cells (cell culture metabolomics) (León et al. 2013; Kalluri et al. 2014) has several advantages like standardization, cost-efficiency, little ethics considerations, and easy integration with other “omics” data. On the other hand, there are also several challenges like mode of harvesting, fast quenching of the metabolism, age dependent proliferation differences, and normalization to cope with (Cuperlović-Culf et al. 2010). In this context, fast quenching of metabolic processes during cell harvesting and data normalization are the two major bottlenecks (Bi et al. 2013; Silva et al. 2013).

Suitable normalization is still an unresolved problem in cell culture metabolomics, although the procedure to correct for biological and/or technical variation is crucial for avoiding erroneous data interpretation. Normalization shall eliminate inter-run variability and batch effects [e.g., by use of internal standards (Ejigu et al. 2013; Kohl et al. 2012; Wang et al. 2012)] as well as biological variation [by relating measured sample data to reliable intrinsic markers for the amount of cells, like sample cell number, wet weight, protein or DNA content, or the total ion current (Cao et al. 2011; Silva et al. 2013; Hutschenreuther et al. 2012)]. For metabolite measurements from cultured cells it is commonly assumed that the metabolite concentration increases with increasing cell number. However, although the linear correlation between the cell number and the total ion current has been demonstrated (Hutschenreuther et al. 2012), a linear correlation of the cell number with the metabolite concentration has so far only been shown for a few selected metabolites. The aspect of linear correlation between metabolite levels and cell number was not the major focus of the cited studies (Silva et al. 2013; Cao et al. 2011; Hutschenreuther et al. 2012) and other comprehensive studies on that topic are yet missing. Therefore, experimental evidence for the general normalization of metabolite concentrations to cell numbers being legitimate is yet very weak.

Direct methods for determination of the cell numbers require either trypsinization or imaging. Trypsinization introduces artifacts into metabolomic data (Bi et al. 2013; Dettmer et al. 2011; Teng et al. 2009), imaging is difficult to apply for cells growing in clumps or 3D culture, and common staining agents often display cytotoxic effects (Bielawski et al. 2001) making downstream applications problematic. Additionally, both methods usually require parallel sampling (Hu et al. 2013) and are labor-intensive, which is unpractical and inefficient, especially in regard to large studies. To guarantee the immediate quenching of the metabolism, adherent cells are often harvested by scraping the cellular layer in organic extraction solvent (Bi et al. 2013; Sapcariu et al. 2014; Dettmer et al. 2011). This approach renders cell counting impossible and as such, many efforts were undertaken to identify a substituting “ideal” reference molecule for data normalization, which is present in the sample used for metabolomics measurements (Cao et al. 2011; Silva et al. 2013). For this reference molecule, a linear correlation between its concentration and the cell number in the sample is desired, regardless of sample preparation and experimental conditions. In some studies, selected metabolites have been used for normalization, which could either be derived from the cells, e.g., the sum of phospholipids (Ruiz-Aracama et al. 2011), or measured in the cell culture supernatant like nutrients and excretion products, e.g., inositol (Cao et al. 2011). The approach seems appealing; however, it has the disadvantage that each “housekeeping metabolite” has to be thoroughly validated for each cell line and each experimental setup. Normalization to the total signal of a metabolite class (Ruiz-Aracama et al. 2011) or the total peak area (Hutschenreuther et al. 2012) is not applicable for the comparison of results from different experiments. Normalization of metabolomics data to the protein content of the sample has been another option (Munger et al. 2006; Dettmer et al. 2011; Silva et al. 2013; Cao et al. 2011). However, protein quantification showed large variations and a low sensitivity at low cell numbers (Silva et al. 2013). Recently, the determination of the DNA concentration was introduced as consistent method for normalization, because the DNA concentration displayed the best linear correlation to the cell number (Silva et al. 2013). Moreover, the DNA can be isolated and quantified directly from metabolomics samples generated by cell scraping in extraction solvent. The method is however not high-throughput feasible, due to a time consuming purification step (Silva et al. 2013).

In the present study, we focused on current challenges in cell culture metabolomics, namely normalization, harvesting, and correlation of metabolite concentrations to the cell number. First, we developed a robust, sensitive, and fast assay for DNA quantification of cell culture derived samples harvested for metabolomics experiments. Second, we analyzed how intracellular metabolites correlate with cell numbers, using a targeted metabolomics approach. Third, we elucidated if our novel DNA quantification assay would be a suitable substitute for cell counting when applied on typical cell culture metabolomics samples, i.e., cells that are harvested by scraping. At last, we investigated the impact of the two different cell harvesting procedures trypsinization and scraping on the concentration of metabolites and on the metabolite-cell number correlation.

## Materials and methods

### Chemicals

Methanol (MeOH; UPLC grade) and isopropanol (HPLC grade) were purchased from AppliChem (Darmstadt, Germany), acetonitrile (HPLC grade) was purchased from Roth (Karlsruhe, Germany), and formic acid (mass spectrometry grade) from Sigma-Aldrich (Hamburg, Germany). Hoechst 33342 was purchased from Life Technologies (Darmstadt, Germany).

### Cell culture

Different human cell lines were selected to cover diverse properties like cell size or tissue origin. All cell lines were maintained at 37 °C and 5 % CO_2_ in a humidified atmosphere and were regularly confirmed to be free of mycoplasma contamination. The identity of all lines was ensured by the cell line authentication service provided by the DSMZ (Braunschweig, Germany). THLE-2 cells, derived from a healthy human liver (ATCC, Wesel, Germany), were cultivated in BEGM (Lonza, Basel, Switzerland) supplemented with 10 % FBS Gold (PAA, Pasching, Austria), 5 ng/mL human recombinant EGF (Life Technologies, Darmstadt, Germany), and 70 ng/mL phosphoethanolamine (Biochrom, Berlin, Germany) according to ATCC guidelines. Cells were cultivated in vessels pre-coated with bovine collagen type I (0.03 mg/mL, BD Biosciences, Heidelberg, Germany), fibronectin (0.01 mg/mL, Sigma Aldrich, Hamburg, Germany), and bovine serum albumin (0.01 mg/mL, Sigma Aldrich, Hamburg, Germany) in BEGM medium. The proximal tubular cell line HK-2, derived from normal human kidney (ATCC, Wesel, Germany), was cultivated in K-SFM (Life Technologies, Darmstadt, Germany) supplemented with 0.05 mg/mL bovine pituitary extract (Life Technologies, Darmstadt, Germany) and 5 ng/mL human recombinant EGF according to ATCC guidelines. The human hepatocarcinoma cell line Hep G2 was purchased from the DSMZ (Braunschweig, Germany) and cultivated in DMEM (Life Technologies, Darmstadt, Germany) supplemented with 10 % FBS Gold. The human preadipocyte cell strain SGBS (Wabitsch et al. 2001; Fischer-Posovszky et al. 2008), kindly provided by Dr. Wabitsch, was cultivated in DMEM F-12 HAM (Sigma-Aldrich, Hamburg, Germany) supplemented with 10 % FBS Gold, 33 µM biotin (Sigma Aldrich, Hamburg, Germany) and 17 µM panthothenate (Sigma Aldrich, Hamburg, Germany).

All cell numbers given in the following refer to the number of cells per sample.

### Harvesting of cells by trypsinization

Cultured cells were washed with warm PBS, incubated for 5 min with 2 mL 0.05 % trypsin containing 0.53 mM EDTA (Life Technologies, Darmstadt, Germany) per 75 cm^3^ flask at 37 °C, and resuspended in 6 mL of the appropriate culture medium. The cells were sedimented at 500 × g and room temperature for 5 (Hep G2 and SGBS) or 10 min (HK-2 and THLE-2). Subsequently, the supernatant was removed and the cell pellet was resuspended in warm PBS. The cells were counted using the Cellometer Auto T4 Plus (PeqLab, Erlangen, Germany), and split into aliquots containing the desired cell number (between 1.0 × 10^4^ and 2.5 × 10^6^) in micro tubes (0.5 mL, Sarstedt, Nümbrecht, Germany). The samples were centrifuged, the supernatants were removed, and the cell pellets were either stored at −80 °C or directly processed for analysis. For analysis, 80 mg glass beads (0.5 mm; VK-05, PeqLab, Erlangen, Germany) and 300 µL of 88 % MeOH precooled on dry ice were added to the tubes and cells were homogenized.

### Harvesting of cells by scraping

Different cell numbers (7.5 × 10^4^ to 5.0 × 10^5^ for THLE-2 and SGBS; 7.5 × 10^4^ to 7.5 × 10^5^ for HK-2, and 2.5 × 10^5^ to 2.5 × 10^6^ for Hep G2) were seeded in 12-well plates in six replicates. Cells were incubated at 37 °C and 5 % CO_2_ for 4 h (THLE-2 and HK-2), 5 h (SGBS) or 16 h (Hep G2). The incubation time was chosen to allow for complete attachment, but short enough to prevent cell proliferation. For the harvest, cells were washed twice with warm PBS, and their metabolism was subsequently quenched by the addition of 200 µL 88 % MeOH, precooled on dry ice. Cells were scraped off the culture vessel using rubber tipped cell scrapers (Sarstedt, Nümbrecht, Germany) and together with the solvent collected in pre-cooled micro tubes containing 80 mg glass beads. The culture well was rinsed with another 100 µL ice-cold 88 % MeOH and the liquid was also transferred to the tube. The samples were stored at −80 °C until further use.

### Homogenization of cells

Cells (supplied with 80 mg glass beads and 300 µL ice-cold 88 % MeOH) were homogenized using a Precellys24 (PeqLab, Erlangen, Germany) at 4–10 °C for two times over 25 s at 5500 rpm. After this, the resulting homogenates were ready to use for the fluorometric DNA quantification as well as for metabolomic analyses.

### Novel fluorometric DNA quantification method

For the development of the fluorometric DNA quantification method, cell homogenates containing 5.0 x 10^5^ of trypsinized cells per sample (Hep G2, SGBS, THLE-2, or HK-2) in 300 µL 88 % MeOH, were used. Pure 88 % MeOH substituted cell homogenates in blank measurements. For the assay, the fluorochrome Hoechst 33342 (10 mg/mL in H_2_O) was diluted in PBS to the according concentrations, as stated below. The indicated amounts of those Hoechst 33342 solutions were put into the wells of a black 96-well plate (F96, Nunc, ThermoFisher, Schwerte, Germany). After brief vortexing of the cell homogenates (samples) or 88 % MeOH (blank), the according aliquots were added to the Hoechst solutions to gain a total volume of 100 µL per well, and the assay components were thoroughly mixed by pipetting. Next, the plate was incubated in the dark for 30 min at room temperature. For quantification of the fluorescence, a GloMax Multi Detection System (Promega, Mannheim, Germany) with an UV filter (λ_Ex_ 365 nm, λ_Em_ 410–460 nm; Promega, Mannheim, Germany) was used. Evaluation of raw data was performed as described below.

In order to determine the dye concentration for optimal assay readout, the Hoechst 33342 stock was diluted to the final concentrations of 0.2, 2.0, 10.0, 20.0, and 30.0 µg/mL. Assays contained 80 µL of the according Hoechst dilutions (corresponding to 0.016, 0.16, 0.8, 1.6 and 2.4 µg dye per assay) and 20 µL of the cell homogenate or 88 % MeOH (blank). For each cell line, six samples per Hoechst 33342 concentration were analyzed in duplicates.

In the next step, the cell homogenate volume required for optimal assay readout was determined. The Hoechst 33342 stock was diluted to the final concentrations of 16.8, 17.8, 18.8, 20.0, 21.3, 22.9, 24.6, 26.7, 29.1, and 32.0 µg/mL. Assays contained 95, 90, 85, 80, 75, 70, 65, 60, 55, and 50 µL of according Hoechst dilutions to get in each case a final amount of 1.6 µg dye per assay. Cell homogenate volumes of 5, 10, 15, 20, 25, 30, 40, and 50 µL were added to the Hoechst solutions to gain a final volume of 100 µL per assay. The final Hoechst 33342 concentration was 20 µg/mL. For each cell line, six samples per Hoechst 33342 concentration were analyzed.

To assess the correlation of cell number to DNA fluorescence intensity, standard curves were generated using the optimized parameters. The assay was thus performed using 80 µL of the optimal Hoechst 33342 solution (20 µg/mL). Cell homogenates with cell numbers ranging from 1.0 × 10^4^ to 1.0 × 10^6^ (THLE-2, HK-2, and SGBS) or to 2.5 × 10^6^ (Hep G2) cells in 300 µL 88 % MeOH were prepared from trypsinized cells. Of these, 20 µL (the optimal cell homogenate volume) each were added to the Hoechst solution. For each cell line, five samples per cell number were analyzed.

### Quantification of metabolites

For targeted metabolomics analysis of cell culture homogenates, the Absolute*IDQ*™ p180 kit (Biocrates Life Sciences, Innsbruck, Austria) was used. The assay is based on tandem mass spectrometry measurements (MS/MS) and allows for the simultaneous quantification of 188 metabolites from different compound classes (21 amino acids, 21 biogenic amines, 40 acylcarnitines, 38 acyl/acyl phosphatidylcholines, 38 acyl/alkyl phosphatidylcholines, 14 lyso-phosphatidylcholines, 15 sphingomyelins, and the sum of hexoses). The metabolites were identified according to MSI Level 1 or 2 (Salek et al. 2013). The complete list of metabolite names, HMDB IDs, and MSI level of identification are given in the Online Resource, Table S-1. The assay has been validated for a number of matrices and showed high precision and reproducibility. The lipids and the hexoses were determined by FIA-MS/MS, while the amino acids and biogenic amines were measured by LC–MS/MS. The method has been described earlier (Zukunft et al. 2013), but was slightly adapted for this study as detailed: 30 µL of the homogenized cell sample were applied manually onto the filter inserts of the 96-well plate provided by the p180 kit by alternation of 10 µL sample with subsequent liquid evaporation.

Sample liquid handling steps were performed by a Hamilton Micro Lab STAR™ robot (Hamilton Bonaduz, Bonaduz, Switzerland) following the manufacturer’s protocol UM-P180. Evaporation steps were performed using a nitrogen evaporator (Ultravap, Porvair Sciences, Leatherhead, Great Britain), and mass spectrometry analyses were done on an API4000 LC–MS/MS system (ABSciex, Darmstadt, Germany) coupled to an Agilent 1200 Series HPLC (Agilent, Böblingen, Germany), and a HTC PAL autosampler (CTC Analytics, Zwingen, Switzerland) controlled by the Analyst 1.5.2 software (ABSciex, Darmstadt, Germany).

On each plate, three plasma samples spiked with different concentrations of reference analytes (QC1-3) and five reference plasma samples were run to serve as quality control and for the evaluation of plate effects, respectively.

### Data analysis

Data obtained from the development of the fluorometric DNA quantification method were plotted using SigmaPlot 12.0 (Systat Software, Erkrath, Germany). SigmaPlot was further used to perform linear regression analysis. The limit of blank (LOB) and the limits of detection (LODs) were calculated according to Armbruster and Pry (Armbruster and Pry 2008). Signal to noise (S/N) ratios were calculated using the Eq. (1) (O’Brien et al. 2005; F. Fan and Wood 2007), where RFU represents relative fluorescence units and SD the standard deviation. A S/N ratio >5 was considered acceptable.

1 $$S/{\text{N ratio = }}\frac{{{\text{RFU }}\left( {{\text{mean}}_{{{\text{sample}}}} } \right){\text{ - RFU }}\left( {{\text{mean}}_{{{\text{blank}}}} } \right)}}{{{\text{SD}}_{{{\text{blank}}}} }}$$

Data evaluation for the quantification of metabolites and quality assessment was performed with the Met*IDQ*™ software, which is part of the Absolute*IDQ*™ p180 kit. Amino acids and biogenic amines were quantified absolutely by using internal standards and calibration curves consisting of 7 calibrators, while acylcarnitines, glycerophospholipids, and hexoses were evaluated semi-quantitatively by using 13 internal standards for lipids and one for the hexoses. All metabolite concentrations are given in µmol/L.

Statistical analysis of metabolomics data was performed using the software R 3.1.2 (R Core Team 2012). Only those metabolites with ≥50 % of samples per cell line displaying a concentration above the LOD (defined by Biocrates for the Absolute*IDQ*™ p180 kit), were considered for further data processing and interpretation. Linear regression analysis was performed to assess the correlation of metabolite concentrations to cell numbers.

To test the applicability of a common standard normalization procedure, which follows the assumption that the slope of the metabolite concentrations, if plotted against the cell number, equals 1, the normalization of the metabolite concentrations of the trypsinized samples to a specific reference cell number (N_c_) was done using the Eq. (2). N_c_ was set to 5.0 × 10^5^. M_m_ represents the measured metabolite concentration and N_d_ the determined cell number. M_cn_ is the metabolite concentration normalized to N_c_.

2 $$M_{\text{cn}} = M_{m} *\left( {\frac{{N_{c} }}{{N_{d} }}} \right)$$

For the comparison of the two harvesting procedures (trypsinization and scraping), we plotted the means of the metabolite concentrations against the means of the determined cell numbers, performed linear regression analysis, and used the obtained metabolite specific linear equations [similar to Eq. (3)] for the calculation of the metabolite concentrations (M_cn_) at the constant reference cell number of 5.0 × 10^5^ cells (N_c_). β_0_ represents the intercept and β_1_ the according slope.

3 $$M_{\text{cn}} = \beta_{0} + \beta_{1} \times {\text{N}}c$$

The testing for statistical significant differences in metabolite concentrations of multiple groups was performed using the non-parametrical Kruskal–Wallis test. For correction of multiple testing, the Bonferroni method was applied. Plots were generated using R 3.1.2 (R Core Team 2012) and the ggplot2 package (Wickham 2009).

## Results and discussion

### Development of the fluorometric DNA quantification method for cell culture metabolomics samples

The quantification of DNA was recently introduced as consistent approach for normalization of metabolomics data from cultured cells (Silva et al. 2013). Since this DNA quantification method is time-consuming and laborious, we developed a robust, sensitive, and fast method for DNA quantification for cell culture derived samples harvested for metabolomics experiments. The principle of the here described DNA quantification assay is the detection of fluorescence after the direct addition of cell homogenates used for metabolomics analyses to a Hoechst 33342 stain [a dye selective for double-stranded DNA (Müller and Gautier 1975)] solution. Assays are performed in a 96-well format. We developed this DNA quantification assay using four different human cell lines, namely Hep G2 (hepatocarcinoma), HK-2 (kidney), THLE-2 (liver), and SGBS (pre-adipocyte), which were harvested by trypsinization.

**Fig. 1:**
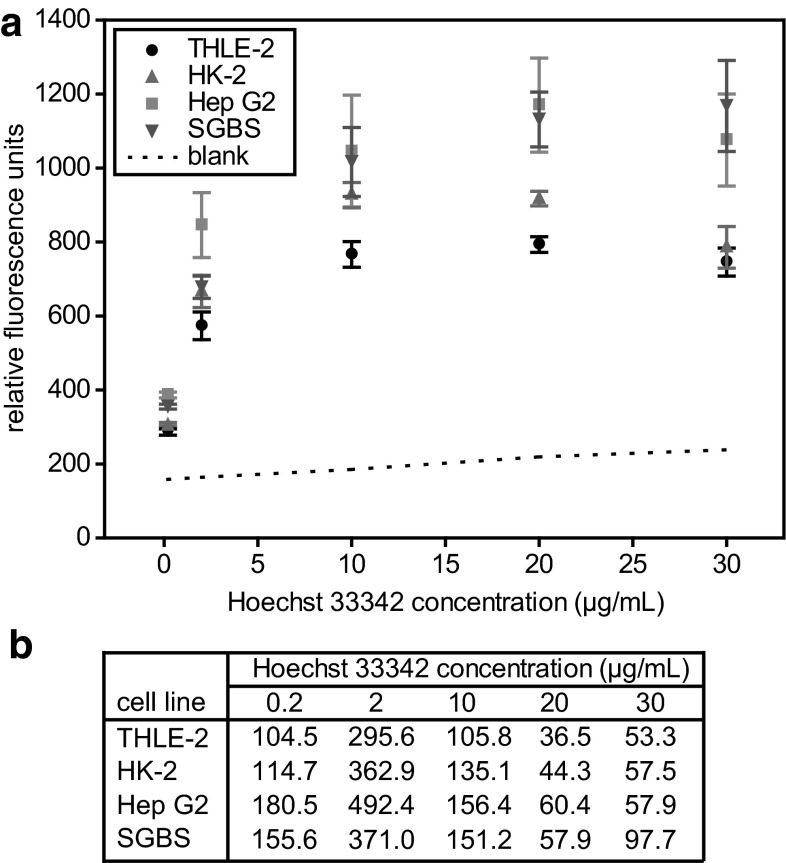
Determination of the optimal Hoechst 33342 concentration for the fluorometric DNA quantification method. 80 µL of differently diluted Hoechst 33342 dye in PBS were mixed with 20 µL of cell homogenate containing 5.0 × 10^5^ cells in 300 µL 88 % MeOH. Blanks contained 20 µL of 88 % MeOH instead of cell homogenate (**a**). Signal to noise ratios for each Hoechst concentration and each cell line (**b**)

The first step during the assay development was the determination of the optimal assay concentration of the fluorescent Hoechst 33342 dye. The manufacturer recommends 0.1–12 µg/mL dye for staining of different cell types. Thus, we tested final dye concentrations ranging from 0.2 to 30 µg/mL. For all cell lines tested, we observed the highest signal intensities and highest S/N ratios at 10–20 µg/mL Hoechst 33342 (Fig. 1). A further increase of the dye concentration to 30 µg/mL led to a slight decrease in fluorescence intensity accompanied by a slight increase in sample variation (Fig. 1). Furthermore, the background signal of the blanks increased with increasing dye concentration, leading to lower S/N ratios with increasing Hoechst dye content (Fig. 1). The cell line independent decrease in fluorescence intensity at high dye concentrations might be explained by self-quenching, a phenomenon described earlier for other fluorescent dyes (Penzkofer and Leupacher 1987; Penzkofer and Lu 1986). Based on the excellent S/N ratios for all cell lines at 20 µg/mL Hoechst 33342 (corresponding to a total amount of 1.6 µg dye per assay), we continued the assay development keeping to this dye concentration.

**Fig. 2:**
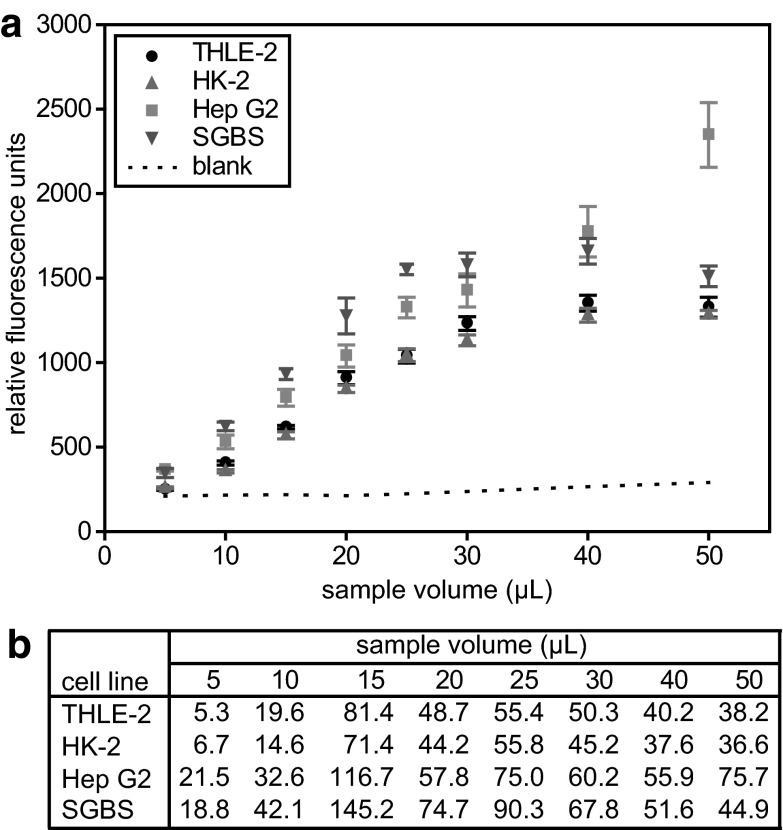
Determination of the optimal cell homogenate volume for the fluorometric DNA quantification method. Different volumes (5–50 µL) of cell homogenates containing 5.0 × 10^5^ cells in 300 µL 88 % MeOH were mixed with different volumes of diluted Hoechst 33342 dye in PBS (50–95 µL; the dye amount per assay was kept constant at 1.6 µg) in a final volume of 100 µL. Blanks contained according volumes of 88 % MeOH instead of cell homogenate (**a**). Signal to noise ratios for each sample volume and each cell line (**b**)

The next step in method development was the determination of the optimal sample volume. To this end, we analyzed different ratios of Hoechst 33342 solution (in PBS) to cell homogenate (in 88 % MeOH). In an assay volume of 100 µL we changed the volume of the Hoechst solution (95–50 µL) but kept the Hoechst 33342 amount with 1.6 µg per assay constant. Cell homogenate volumes ranged from 5 to 50 µL. For all cell lines tested, we observed an increase of the fluorescence signal with increasing homogenate volume. However, three out of four cell lines, namely SGBS, THLE-2, and HK-2, displayed signal saturation (Fig. 2) at cell homogenate volumes higher than 25–30 µL, whereas Hep G2 cell signals reached no plateau (Fig. 2). A possible explanation for this observation might be that Hep G2 homogenates exert less matrix effect related quenching of the fluorescent signal due to the smaller cell volume, which might correlate with a lower amount of potential interfering intracellular compounds. With increasing homogenate volume, also the measured background of the blanks increased, diminishing the S/N ratios. Considering the necessity to use as little sample as possible but as much as necessary to obtain an optimal S/N ratio, we decided on the optimal sample volume being 20 µL.

We performed all subsequent measurements with the following fixed assay composition: assays contained 80 µL of a 20 µg/mL Hoechst 33324 solution (in PBS; final Hoechst 33342 content of 1.6 µg per assay) and 20 µL of cell culture homogenate (in 88 % MeOH) in a total assay volume of 100 µL.

**Fig. 3:**
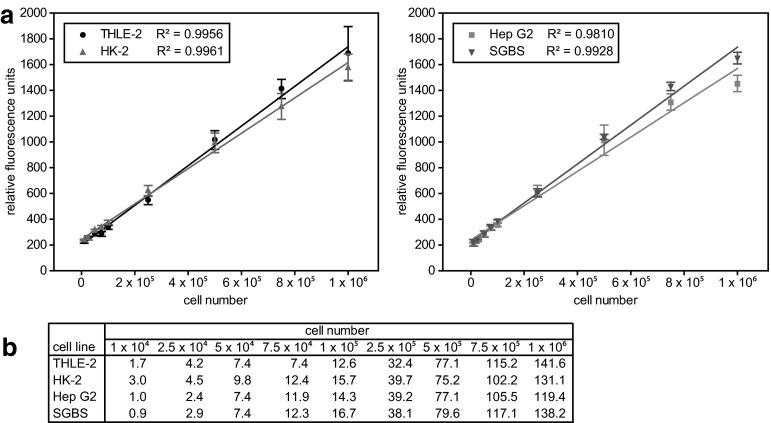
Correlation of Hoechst 33342 fluorescence intensity with cell number. 20 µL of cell homogenates, containing different amounts of cells per sample (1.0 × 10^4^ to 1.0 × 10^6^ in 300 µL 88 % MeOH), were added to 80 µL of a Hoechst 33342 solution (20 µg/mL in PBS). Blank measurements were carried out using 20 µL of 88 % MeOH instead of cell homogenate. Coefficient of determination (R^2^) for each cell line is given in the legend (**a**). Signal to noise ratio for each cell number and each cell line (**b**)

To assess the correlation of cell number to DNA fluorescence intensity, we recorded a standard curve for each cell line. Therefore, we used homogenates with cell numbers ranging from 1.0 × 10^4^ to 1.0 × 10^6^ per sample. For all four cell lines tested, we observed excellent linear correlation of the relative fluorescence units (RFUs) with the cell numbers over two orders of magnitude (Fig. 3). The same observation was made for other cell lines, namely HEK293, Hepa1-6, COS-1, HeLa, and 3T3-L1 (R^2^: 0.9889–0.9997; data not shown). The limit of blank (LOB) was calculated to be 225.1 RFUs, while the limit of detection (LOD) was calculated for each cell line individually. The LODs for THLE-2, HK-2, Hep G2, and SGBS were 244.9, 233.8, 230.4, and 266.8 RFUs, respectively. These LODs corresponded to 2.5 × 10^4^ (THLE-2), 1.0 × 10^4^ (HK-2), 2.5 × 10^4^ (Hep G2), and 5.0 × 10^4^ (SGBS) cells per 300 µL sample. Considering a S/N ratio of larger than 5 as acceptable, the lower limit of quantification (LOQ) was found to be at 5.0 × 10^4^ cells per sample for all cell lines analyzed in this study. In some cases we noticed a slight tendency to reach a signal saturation of fluorescence intensity at 1.0 × 10^6^ cells (Fig. 3). However, the linear range of our assay was found to be within 5.0 × 10^4^ and 1.0 × 10^6^ cells per 300 µL sample for THLE-2, HK-2, and SGBS cells (R^2^: 0.9928 - 0.9961) and within 5.0 × 10^4^ and 2.5 × 10^6^ cells per 300 µL sample for Hep G2 cells (R^2^: 0.9713).

It was recently shown that the DNA concentration determined in cell culture metabolomics samples has an excellent linear correlation with the cell number of these samples (Silva et al. 2013). However, the authors applied a complicated and time-consuming workflow by first isolating and purifying genomic DNA from the samples and in a second step quantifying the DNA spectrophotometrically. In comparison, our fluorometric method for DNA quantification has only little time and material requirements, since the method is fast and needs only a DNA-binding dye, multi-well plates, and a plate reader. Furthermore, the method has a low error-proneness, a wide linear range and is quite robust. For instance, cell numbers can be determined regardless of cell size, cell line, and tissue of origin (Fig. 3). Also, the methanol content of the homogenization (extraction) solvent, within a range of 60–100 %, does not have a significant impact on the obtained signal (data not shown). Additionally, the method provides a large flexibility regarding e.g., sample volume or dye concentration, as can be concluded from the optimization experiments. However, as with every method, there are certain limitations to consider. For instance, compound induced ploidy changes in cells might have an impact on the measured fluorescence intensity. To circumvent this effect, we would recommend to record standard curves in any new experimental cell culture setup. Furthermore, our method fails at very low cell numbers due to insufficient sensitivity. However, as the method is very flexible this issue might be overcome by adjusting single assay parameters (e.g., the Hoechst 33342 concentration). In conclusion, the use of such a universal molecule like DNA for normalization of metabolomics from cell culture in combination with a robust, but still flexible quantification method entails only little validation work when applied to different cell lines.

### Correlation between cell number and metabolite concentration

For the normalization of metabolomics data from cells, it is usually assumed that an increase of cell number gains an increase of metabolite signals, and the ideal case would be a linear correlation. However, to the best of our knowledge, this assumption was not yet proven systematically with a broad panel of quantified metabolites and cell lines. For the cell lines Hep G2, THLE-2, SGBS, and HK-2 we therefore assessed the correlation between cell number and metabolite concentration by quantifying the metabolite levels of different amounts of trypsinized cells. We selected cell numbers lying within the linear range of the DNA standard curves. We determined metabolite levels using a validated targeted metabolomics kit, the Absolute*IDQ™* p180 kit from Biocrates. Although this targeted metabolomics approach allows for the parallel quantification of a limited panel of metabolites (188 metabolites from six different compound classes (amino acids, biogenic amines, acylcarnitines, phospho- and sphingolipids as well as the sum of hexoses)), the kit was chosen for two good reasons: first, it contains the largest set of metabolites quantifiable at the same time, and second, it provides absolute concentrations, which is essential to perform correlation analyses. Only metabolites which passed the quality threshold criterion (≥50 % of samples per cell line displaying concentrations above the LOD) were taken into account for further calculations and evaluations. These measures were taken to minimize the distortion of the results due to technical limitations of the analysis. Depending on the cell line, 85–114 metabolites were found to be above the LOD (Table 1). The performance of a linear regression analysis showed that more than 90 % of these metabolites displayed an excellent linear correlation (R^2^ ≥ 0.9) between concentration and cell number (Online Resource, Fig. S-1), and more than 50 % surpassed even an R^2^ value of 0.99. However, the slopes of the regression lines were found to be metabolite and cell line dependent (Online Resource, Fig. S-3, Table S-2). The different rates of increase might originate from matrix and analyte dependent differences in ionization properties and ion suppression as well as from cell line specific utilization of metabolic pathways (Jain et al. 2012; Neermann and Wagner 1996).

**Table 1:** Quality of linear correlation between metabolite concentration and cell number

	% of Metabolites with coefficients of determination (R^2^) > 0.9	
Cell line	Trypsinized cell samples	Scraped cell samples
THLE-2	96 % (n = 114)	93 % (n = 94)
Hep G2	94 % (n = 94)	93 % (n = 95)
HK-2	91 % (n = 85)	84 % (n = 51)
SGBS	97 % (n = 110)	82 % (n = 114)

Different numbers of cells of each cell line were harvested by trypsinization or scraping and targeted metabolomics was performed. Exact cell numbers were determined directly by counting (trypsinized cells) or indirectly by our new fluorometric DNA quantification method (scraped cells). Linear correlation between cell numbers and metabolite concentrations was performed including only the metabolites passing the quality threshold criterion (≥50 % of samples per cell line displaying concentrations above the LOD) (numbers given in brackets)

Around 10 % of metabolites which passed the quality threshold criterion did not exhibit sufficient linearity for normalization purposes (Online Resource, Fig. S-2), in most of the cases probably due to concentration values very close to the LOD, as shown exemplarily for the acylcarnitine C16:1 (Online Resource, Fig. S-4). In addition, many of the affected metabolites were part of the lipid panel of the Biocrates Absolute*IDQ™* p180 kit. The lipids are measured using only a semi-quantitative approach (no individually matching internal standard for every single metabolite, but one internal standard for several similar metabolites). Hence, the concentration values of these metabolites are more prone to evaluation errors, because metabolite and internal standard might show different matrix effects or ionization efficiencies.

Published data on correlation of metabolite concentrations to cell numbers are rare and our data thus overlap only with those for one metabolite, namely glutamic acid. Glutamic acid was found to correlate linearly with the cell number in a LC–MS (Silva et al. 2013) and a GC-TOF–MS (Cao et al. 2011) approach supporting our observations. The other metabolites analyzed in these studies (Cao et al. 2011; Silva et al. 2013) were organic compounds, which were not included in our method. However, those compounds showed as well linear correlation with cell number leading to the assumption that the linear correlation behavior holds true for most metabolites. On the other hand, metabolites of different chemical classes as well as metabolite analyses techniques are so diverse that a reliable prediction of metabolite behavior in analytics is difficult.

All in all, the excellent correlation of most metabolite concentrations to the cell number over different metabolic classes shown in our and in previous studies demonstrates that the assumption of increasing metabolite levels with increasing cell numbers holds true. Further, this observation underlines the eligibility of data normalization to the cell number.

### Applicability of the fluorometric DNA quantification as normalization method for cell culture metabolomics

After having shown that both the fluorometric DNA signal and the metabolite concentration are linearly correlating with the cell number, we assessed the applicability of the indirect cell counting, i.e., the fluorometric DNA quantification, for cell culture metabolomics normalization. We harvested cells according to our standard cell culture procedure for metabolomics sample generation by scraping the cell layer in pre-cooled extraction solvent. We employed cell numbers within the range of 7.5 × 10^4^ to 2.5 × 10^6^ cells. Metabolites were quantified as before by targeted metabolomics and depending on the cell line, 51–114 metabolites were found to be above the LOD (Table 1). These metabolites were used for further analysis. In parallel, the cell numbers contained in the samples were determined indirectly using our fluorometric DNA quantification method and calculated by means of standard curves. Linear correlation analysis between the metabolite concentration and the indirectly measured cell numbers were performed. For THLE-2 and Hep G2 cells, more than 90 %, and for SGBS and HK-2 cells, more than 80 % of the measured metabolites above LOD displayed an R^2^ value above 0.9 (Table 1). Additionally, in all cell lines, more than 50 % of all metabolites showed an R^2^ value larger than 0.95. In conclusion, we obtained linear correlations of metabolite concentrations with the cell numbers in samples harvested by scraping. Since we also obtained linear correlations of metabolite concentrations with cell number in samples harvested by trypsinization, we conclude that the fluorescent method for DNA quantification is applicable for normalization of cell culture derived samples in metabolomics analyses.

Normalization of metabolomics data to parameters such as cell number, DNA, or protein content, and subsequent statistical analysis are common procedures in data processing (León et al. 2013; Hutschenreuther et al. 2012; Bi et al. 2013; Silva et al. 2013) and of vital importance for data interpretation (Silva et al. 2013; Cuperlović-Culf et al. 2010). However, massive efforts are made to eliminate bias in data analysis (Broadhurst and Kell 2006; Burton et al. 2008; Wang et al. 2012), but up to now, the question whether normalization of the data can introduce a bias and false positives is strongly underrepresented in the literature. To address one aspect of this issue, we elucidated whether a significant difference in metabolite concentrations would appear between samples measured at different cell numbers but normalized to one fixed reference cell number. To this end, normalized metabolite concentrations of all samples with different numbers of scraped cells were calculated according to Eq. (2) using 5.0 × 10^5^ cells as reference cell number. For SGBS, THLE-2, Hep G2, and HK-2 cells 87, 82, 60, and 61 % of all metabolites passing the quality threshold criterion (≥50 % of samples per cell line displaying concentrations above the LOD), respectively, displayed constant values (Online Resource, Fig. S-5). The observed differences between the cell lines resulted most likely from the different cell specific metabolic profiles. For example, of all cell lines HK-2 showed the lowest number of metabolites meeting the quality threshold criterion (Table 1), and in consequence, only a low number of linearly behaving metabolites could be found. Additionally, many measured metabolite concentrations were in close vicinity to the LOD. Such low values are usually overestimated in the normalization process leading mostly to a non-linearity of the respective metabolites.

Overall, the results indicate that the concentration of the majority of metabolites can be measured at any cell amount (within the linear range of the method and when the metabolite concentrations lie above the LOD) and their concentrations can be extrapolated in a linear fashion to a reference cell number. However, the observation that 13–40 % of all metabolites showed significant concentration differences after normalization to one fixed cell number, underlines the importance of a thorough validation of normalization procedures in study design and data processing.

### Impact of the harvesting method on the metabolite concentrations

A vital prerequisite for conducting reliable metabolomics is the immediate quenching of metabolic processes (León et al. 2013; Dettmer et al. 2011; Bi et al. 2013; T. W.-M. Fan 2012) at sample collection. In order to meet this requirement, scraping of cells in an ice-cold extraction solvent consisting of a mixture of organic and aqueous components is currently the method applied most frequently (Hutschenreuther et al. 2012; Teng et al. 2009; Dettmer et al. 2011; Bi et al. 2013). However, trypsinization is the standard cell culture procedure for the detachment of adherent cells and allows for convenient cell counting. Unfortunately, it is less suitable for metabolomics analysis, because the metabolism is not quenched immediately at cell harvest and a decrease in metabolite concentration of some small and polar compounds (trypsin leakage) was reported (Teng et al. 2009; Dettmer et al. 2011; Bi et al. 2013). To elucidate the impact of enzymatic and mechanical sample preparation on a broader panel of metabolite classes in targeted metabolomics analysis, we compared the metabolite concentrations of trypsinized and scraped cells. In case of the trypsinized cells, the precise cell number was determined after harvesting by counting and homogenates which contained the reference cell number of 5.0 × 10^5^ cells were used. In case of the scraping approach, 5.0 × 10^5^ cells per well were seeded, incubated in medium until full attachment was achieved but before proliferation started, and then harvested by scraping. As seeding and scraping might lead to a loss of cells, the exact cell numbers in the homogenates were determined using the fluorescence-based DNA quantification method. Although identical cell numbers were used for both harvesting approaches, the cell numbers in the scraped samples of THLE-2, HK-2, and Hep G2 were slightly lower, which is probably due to the stress during passaging prior to seeding (data not shown). Thus, the obtained metabolite concentrations were normalized to 5.0 × 10^5^ cells for the data comparison. As discussed previously, the standard normalization procedure, which assumes a slope of 1 if the metabolite concentrations are plotted against the cell numbers, was not applicable for all metabolites. Therefore, linear regression analysis was performed for each metabolite [see Eq. (3)] and the resulting parameters (slope and intercept) were used for normalization. The impact of the harvesting method turned out to be highly specific in regard to metabolite class as well as to cell line (Online Resource, Fig. S-6). Acylcarnitine levels were not affected by the harvesting method, but we observed substantial differences between trypsinized and scraped cells for amino acids, biogenic amines, lyso-phosphatidylcholines, phosphatidylcholines, and sphingomyelins. THLE-2 and SGBS cells contained considerably lower and Hep G2 cells slightly lower mean levels of amino acids and biogenic amines in trypsinized than in scraped cell samples (Table 2). Different to the other three cell lines, HK-2 cells showed higher amino acid and biogenic amine concentrations in trypsinized than scraped cells. Interestingly, phosphatidylcholines, which are major constituents of cellular membranes (Colbeau et al. 1971), and their degradation products, the lyso-phosphatidylcholines, predominantly displayed strongly elevated levels in samples collected by trypsinization. Regarding sphingomyelins, trypsinized THLE-2, Hep G2, and HK-2 cells showed 1.5–3.1 fold increased levels compared to the scraped cells. In contrast, only 60 % of the total sphingomyelin concentration in trypsinized SGBS cells was present when compared to scraped cells (Table 2).

**Table 2:** Fold changes in metabolite concentrations when trypsinized cell homogenates were compared to scraped cell homogenates (5.0 × 10^5^ cells per sample)

	Mean fold changes ± SD				
Cell line	Acylcarnitines	Amino acids and biogenic amines	Lyso-phosphatidylcholines	Phosphatidylcholines	Sphingomyelins
THLE-2	1.05 ± 0.30	0.68 ± 0.47	2.39 ± 0.08	1.60 ± 0.38	1.53 ± 0.30
Hep G2	NA^a^	0.91 ± 1.14	1.10 ± 0.23	2.46 ± 1.03	2.21 ± 0.85
HK-2	NA^a^	1.73 ± 1.03	NA^a^	2.69 ± 0.64	3.13 ± 0.71
SGBS	0.96^b^	0.39 ± 0.19	1.23 ± 0.18	0.70 ± 0.16	0.60 ± 0.20

^a^ *NA* not applicable—Either no metabolites above the LOD were detected, or those metabolites above the LOD were only detected in samples derived from one harvesting method, but not in the other

^b^ Only a single metabolite above LOD was detected for this metabolite class

Substantial loss of intracellular metabolites in trypsinized cell samples has already been reported for different adherent cell lines (Teng et al. 2009; Dettmer et al. 2011; Bi et al. 2013). These studies focused on small and polar metabolites like amino acids, tricarboxylic acid cycle intermediates, nucleobases, and sugars. The authors discussed metabolite secretion (Teng et al. 2009), passive diffusion due to different osmotic strength of the applied solutions (Teng et al. 2009), metabolite fluctuations caused by rapid turnover rates in response to changes in the cell environment and cell morphology (Teng et al. 2009; Bi et al. 2013), and leakage of metabolites through the plasma membrane (Dettmer et al. 2011) as possible reasons for the depletion of intracellular metabolites upon trypsinization of cells. Bi et al. also noted that the metabolic leakage most likely depends on the cell type (Bi et al. 2013). Our data strongly underline this opinion, since we observed that the effect of trypsin on the intracellular metabolites is not only metabolite class dependent but also cell line dependent. Regarding amino acid and biogenic amine concentrations, we found them to be considerably lower in trypsinized samples of THLE-2, Hep G2, and SGBS cells, which is in agreement with previously published work on other cell lines (Dettmer et al. 2011; Bi et al. 2013; Teng et al. 2009). An explanation might be metabolite leakage due to enzymatic decomposition of the cellular membrane and PBS washing steps during enzymatic harvest. Why HK-2 cell homogenates showed elevated amino acid and biogenic amine levels in trypsinized samples can unfortunately not yet be explained. For lyso-phosphatidylcholines, phosphatidylcholines, and sphingomyelins we mostly observed increased levels in trypsinized samples (Table 2). However, trypsinized SGBS cells were found to be the exception in displaying slightly decreased lipid levels, thereby further underlining the cell line dependency of the impact of the applied harvesting method.

## Concluding remarks

We have developed a fluorescence-based DNA quantification method for the determination of cell numbers in metabolomics samples. This assay is robust, allows for fast quantification of the DNA content, is easier and faster than currently used alternative methods, and facilitates normalization procedures in cell culture metabolomics. Furthermore, we found the metabolite concentrations of most metabolites from different classes to be positively correlated with the cell number in a linear fashion, which provides the eligibility of data normalization to the cell number. We also showed that the impact of the cell harvesting protocol is highly dependent on the metabolite class and the cell line. Our observations that a small portion of metabolites showed no linear correlation to the cell number as well as the cell line specific impact of the harvesting procedure on the metabolite concentrations, underline the importance of thorough optimization, standardization, and validation of cell culture metabolomics experiments.

## Electronic supplementary material

Below is the link to the electronic supplementary material.

Supplementary material 1 (PDF 831 kb)
