# Supplementary material for: Removing the bottlenecks of cell culture metabolomics: fast normalization procedure, correlation of metabolites to cell number, and impact of the cell harvesting method
Source: Metabolomics. 2016 Sep 15;12(10):151. doi: 10.1007/s11306-016-1104-8 (PMC5025493; doi:10.1007/s11306-016-1104-8)
Supplement: Supplementary file 1 — Supplementary material 1 (PDF 831 kb) [file 11306_2016_1104_MOESM1_ESM.pdf]

Metabolomics

Removing the bottlenecks of cell culture metabolomics: fast normalization procedure, correlation of metabolites to cell number, and impact of the cell harvesting method

Caroline Muschet [†], *Gabriele Möller* [†], *Cornelia Prehn* [†], *Martin Hrabě de Angelis* [†,§,$], *Jerzy Adamski* [†,$,§], *and Janina Tokarz* [†,*]

[†] Helmholtz Zentrum München, German Research Center for Environmental Health, Institute of Experimental Genetics, Genome Analysis Center, Ingolstaedter Landstrasse 1, 85764 Neuherberg, Germany
[§] Lehrstuhl für Experimentelle Genetik, Technische Universität München, 85350 Freising-Weihenstephan, Germany
[$] German Center for Diabetes Research (DZD), 85764 Neuherberg, Germany

Corresponding author: Janina Tokarz, Helmholtz Zentrum München, German Research Center for Environmental Health, Institute of Experimental Genetics, Genome Analysis Center, Ingolstaedter Landstrasse 1, 85764 Neuherberg, Germany
Voice: +49-89-3187-3233, Fax: +49-89-3187-3225, e-mail: janina.tokarz@helmholtz-muenchen.de

Table of contents

Fig. S-1 Histograms of the R^2 distributions indicate excellent correlations of the metabolite concentration with the cell number for the majority of metabolites and both harvesting methods (trypsinization and scraping).

Fig. S-2 Distribution of the R^2 coefficients after linear correlation of the cell number to the metabolite concentration for all cell lines and all metabolites show only few weakly correlated metabolites.

Fig. S-3 Correlation of cell number and metabolite concentration for representative metabolites in four cell lines.

Fig. S-4 The acylcarnitine C16:1 shows no linear correlation to the cell number in all cell lines.

Fig. S-5 Normalization of metabolites from samples with different cell numbers to the fixed reference cell number of 5.0×10^5 cells per sample.

Fig. S-6 The fold changes of metabolite concentrations of trypsinized compared to scraped cells demonstrate that the harvesting impacts the metabolite concentration in a cell type and metabolite class dependent manner.

Table S-1 List of measured metabolites with their abbreviations, names, metabolite classes, HMDB IDs, and MSI levels.

Table S-2 List of the slopes of the linear regression between cell number and analyte concentration.

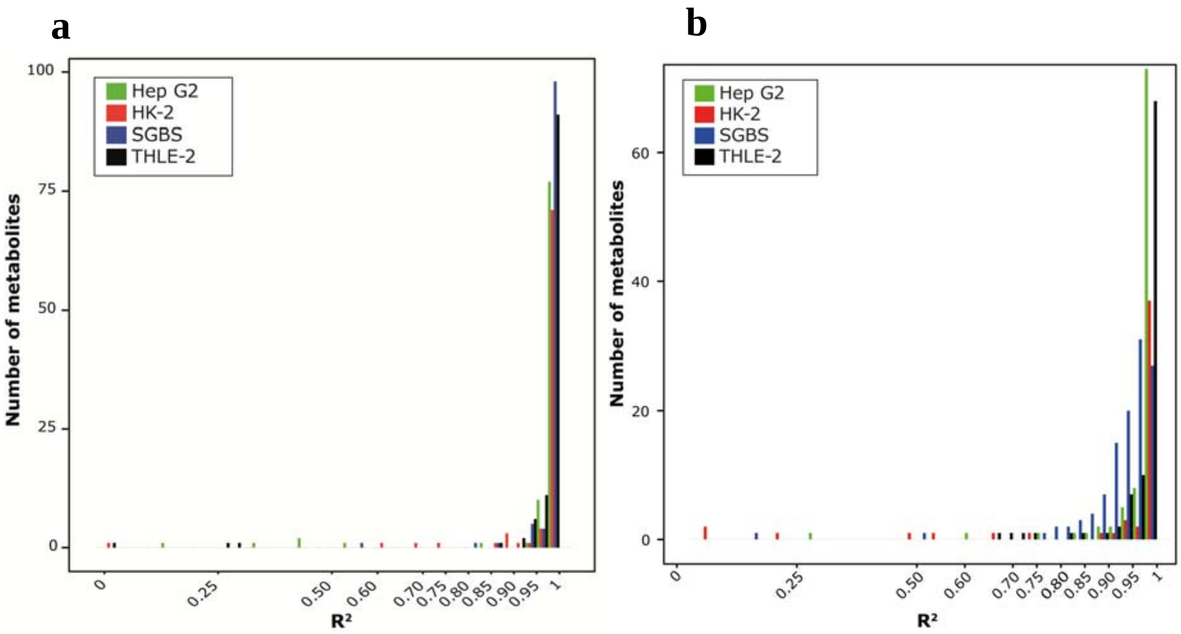

Fig. S-1 Histograms of the R^2 distributions indicate excellent correlations of the metabolite concentrations with the cell number for the majority of metabolites and both harvesting methods (trypsinization and scraping).

We harvested different cell amounts within the linear range of the DNA quantification method by either trypsinization (a) or scraping (b) and performed targeted metabolomics as described in the materials and methods section. Linear correlation between the cell number and the metabolite concentration was analyzed using the metabolites above the LOD. The distribution of the calculated coefficients of determination (R^2 coefficients) is depicted in the histograms.

Metabolomics

a

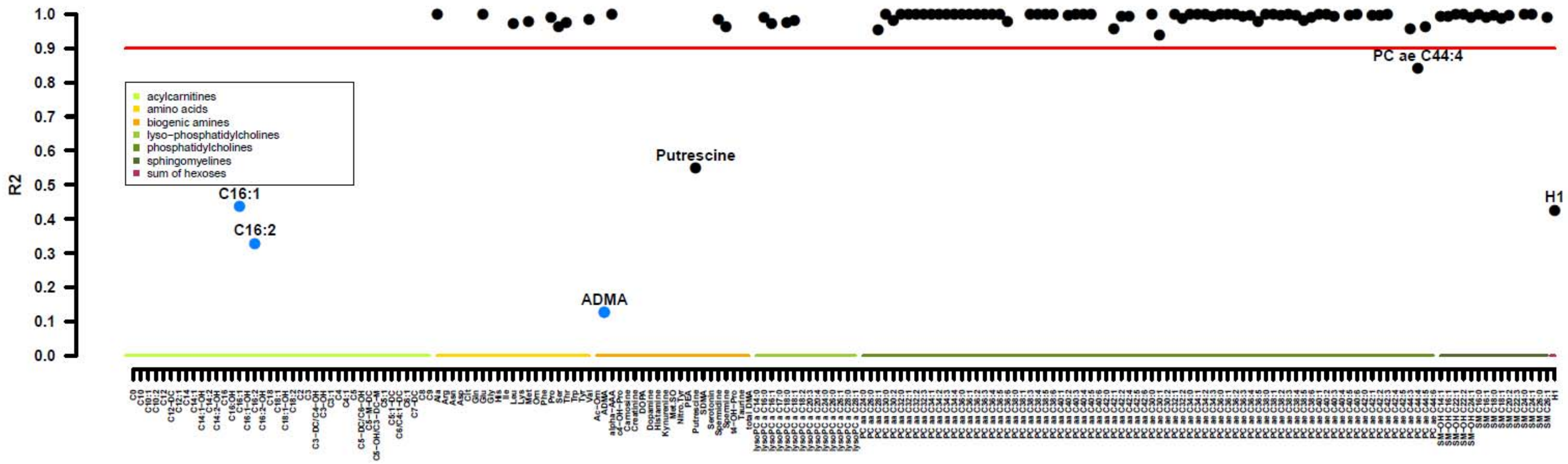

b

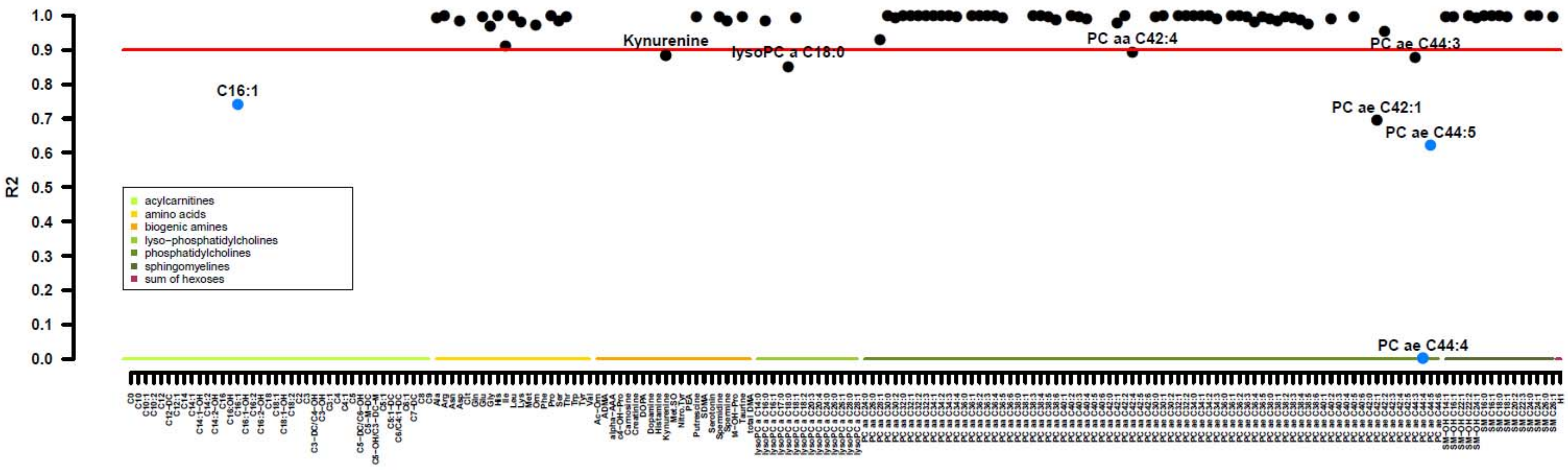

Metabolomics

c

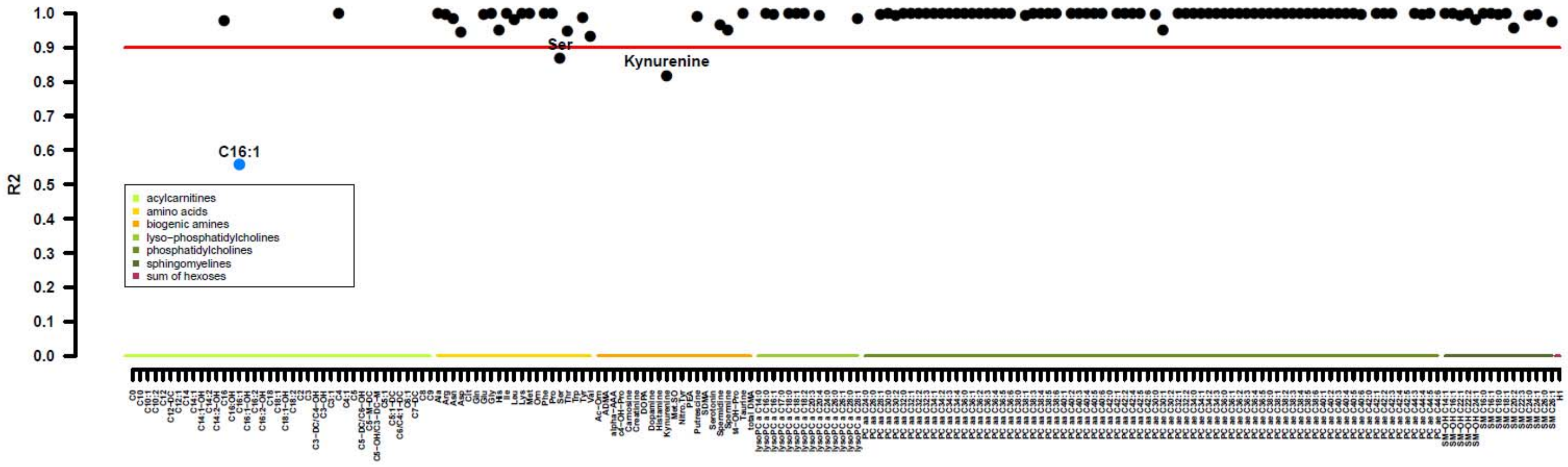

d

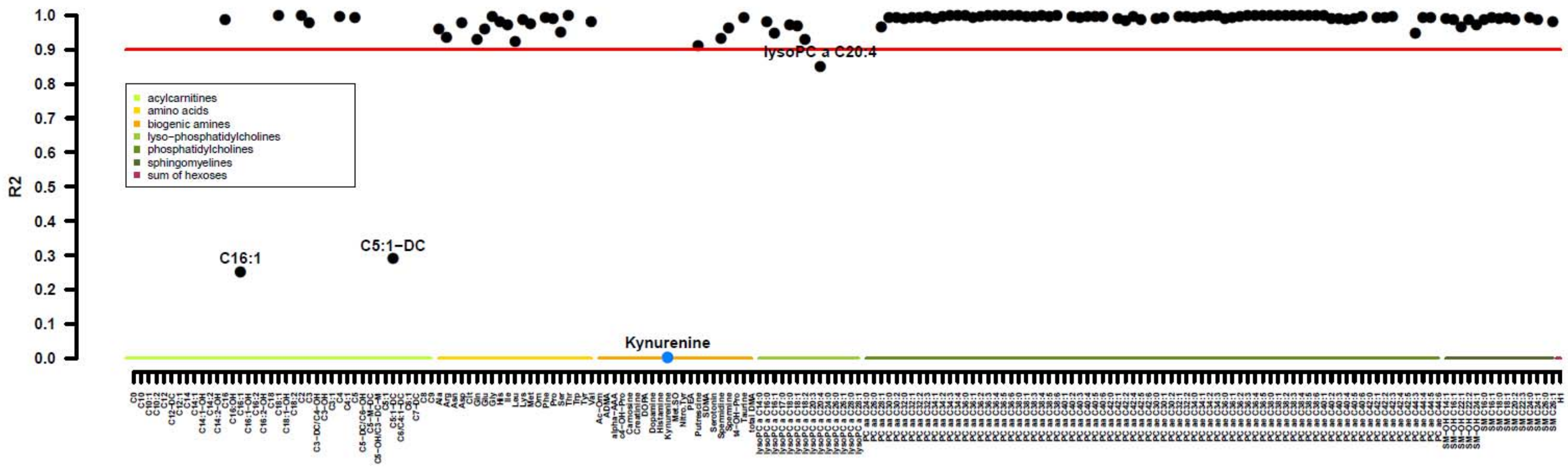

Fig. S-2 The distribution of the R^2 coefficients after linear correlation of the cell number to the metabolite concentration for all cell lines and all metabolites shows only few weakly correlating metabolites.

We harvested different cell amounts containing cell numbers within the linear range of the DNA quantification method by trypsinization and performed targeted metabolomics as described in the materials and methods section. Linear correlation between the cell number and the metabolite concentration was analyzed. The calculated coefficients of determination (R^2 coefficients) are plotted as dots for each metabolite. Identities of the single metabolites are denoted on the x axis and metabolite classes are grouped as indicated by colored bars. The horizontal red line marks the R^2 value of 0.9. Black dots indicate a correlation between cell number and metabolite concentration with a positive slope, whereas blue dots represent a correlation with a negative slope. If no dot is presented for a given metabolite, this metabolite was found to be below the LOD. The results for Hep G2 (a), HK-2 (b), SGBS (c), and THLE-2 (d) are shown.

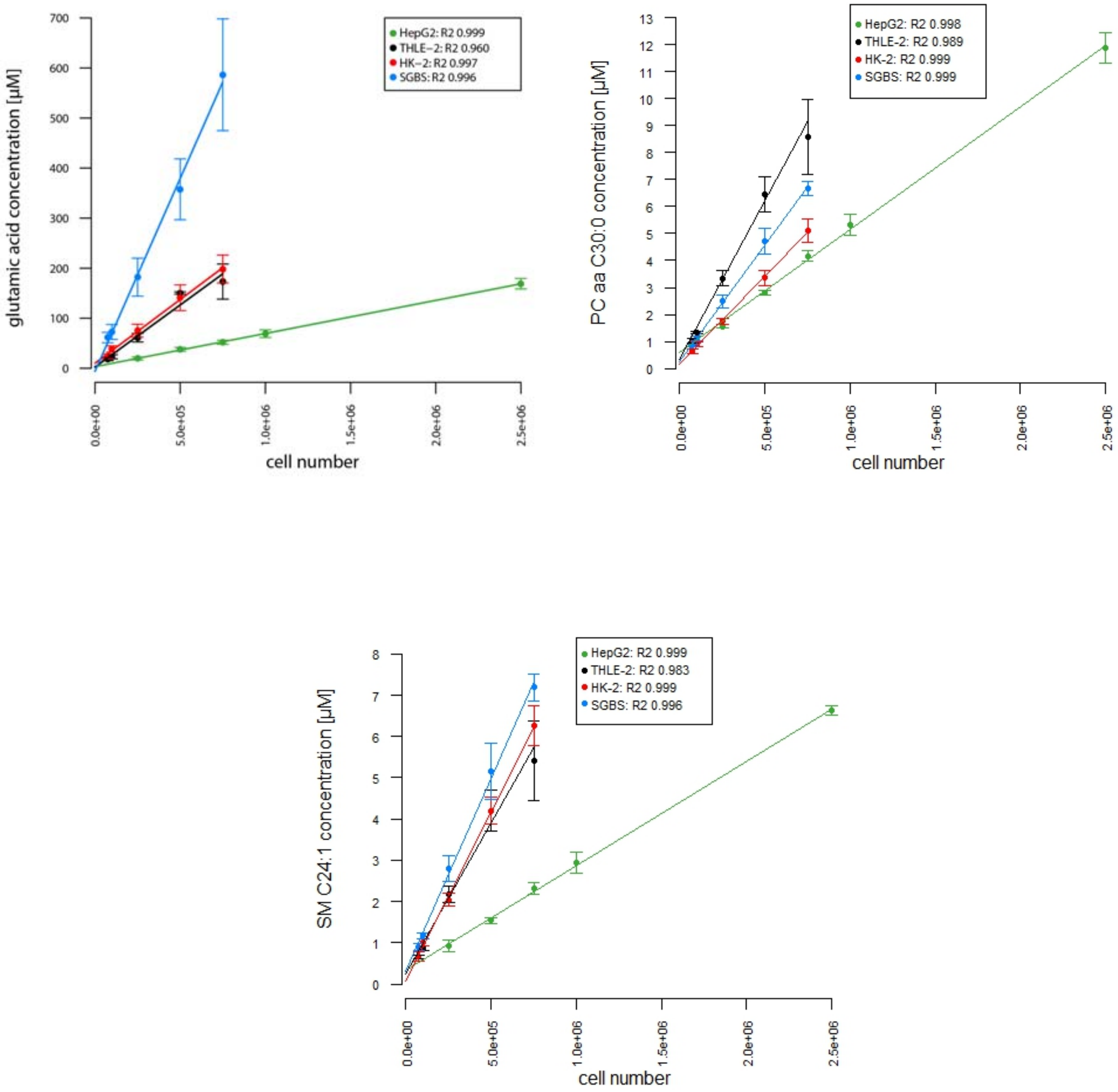

Fig. S-3 Correlation of cell number and metabolite concentration for representative metabolites in four cell lines.

We harvested different cell amounts containing cell numbers within the linear range of the DNA quantification method by trypsinization and performed targeted metabolomics as described in the materials and methods section. Correlation between the cell number and the metabolite concentration was analyzed. The figure shows the linear correlations of a selected panel of metabolites, which are representatives for the respective metabolite classes. The slope of the linear regression and the absolute metabolite concentrations are cell line and analyte dependent. The slopes for all metabolites with concentrations above the LOD in all cell lines are listed in Table S-2.

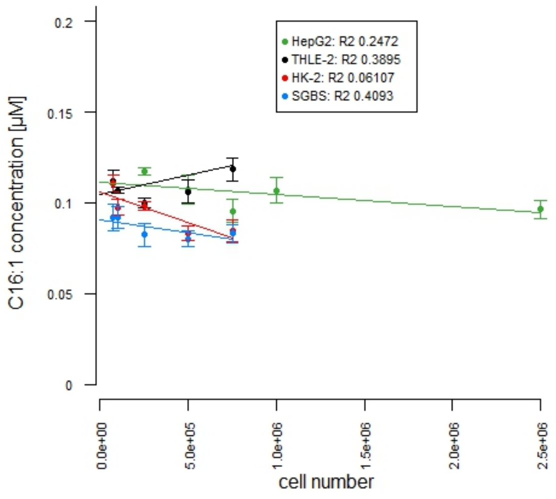

Fig. S-4 The acylcarnitine C16:1 shows no linear correlation to the cell number in all cell lines. Different numbers of cells were harvested via trypsinization and targeted metabolomics was performed as described in the materials and methods section. Correlation of the analyte concentration to the cell number was analyzed. C16:1 does not exhibit a linear correlation between cell number and metabolite concentration in any of the tested cell lines. This might be due to technical and biological limits. First, C16:1 can only be determined semi-quantitatively, because an individually matching internal standard for C16:1 is not included in the Biocrates Absolute*IDQ*™ p180 kit. Second, the measured metabolite concentrations are very close to the LOD of the metabolite (0.06 µM).

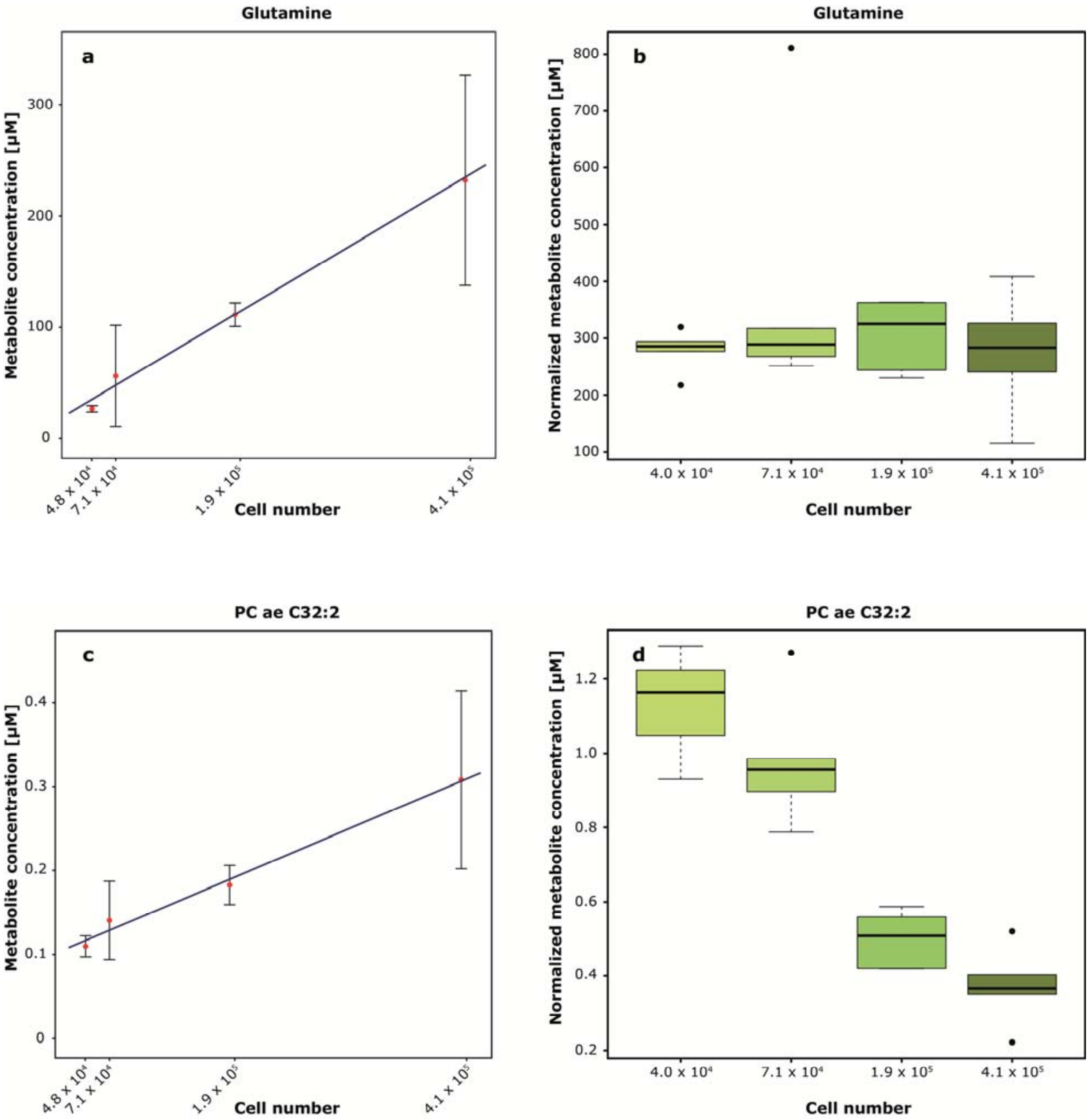

Fig. S-5 Normalization of metabolites from samples with different cell numbers to the fixed reference cell number of 5.0 x 10^5 cells per sample.

We harvested different cell numbers of all cell lines within the linear range of the DNA quantification method via scraping and performed targeted metabolomics as described above and in the materials and methods section. Only metabolites above the LOD were used for the analysis. This figure shows exemplary data derived from THLE-2 cells. Panels a and c show the linear correlations between the cell number and the metabolite concentration for glutamine and PC ae C32:2. Panels b and d show the normalized metabolite concentrations to the reference cell number of 5.0 x 10^5 cells (using equation (2))

of glutamine and PC ae C32:2. Significant differences between the calculated normalized concentrations were tested by using the non-parametrical Kruskal-Wallis test with Bonferroni correction for multiple testing (threshold for THLE-2 cells is 5.3×10^{-4}). Measured data for glutamine could be successfully normalized to the reference cell number (p-value 0.78), whereas PC ae C32:2 showed a significant change (p-value 2.4×10^{-4}) in its normalized concentrations.

The data for glutamine from THLE-2 cells are representative for 60% to 87% of all measured metabolites from all cell lines.

Metabolomics

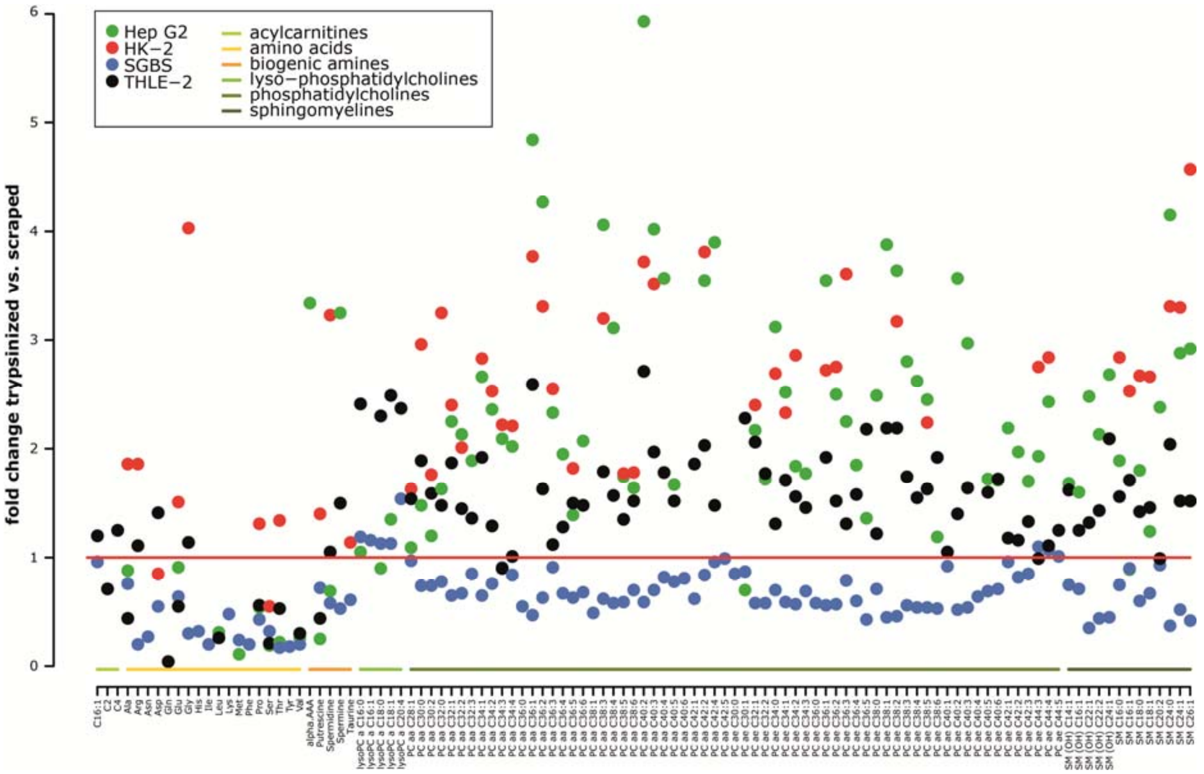

Fig. S-6 The fold changes of metabolite concentrations of trypsinized compared to scraped cells demonstrate that the harvesting impacts the metabolite concentration in a cell type and metabolite class dependent manner.

We harvested 5.0 x 10^5 cells (THLE-2, Hep G2, HK-2, and SGBS) by trypsinization. For the harvest by scraping, we seeded 5.0 x 10^5 cells and scraped the cells after sufficient adherence time. We performed targeted metabolomics as described in the materials and methods section. Only metabolites above the LOD were used for further analysis. The cell number in the scraped samples was determined by our new fluorescence-based DNA quantification method. Since the measured cell numbers of the scraped samples differed from the seeded ones, normalization was required. Therefore, linear regression analysis was performed and metabolite concentrations, corresponding to the reference cell number of 5.0 x 10^5 cells, were calculated using equation (3). Finally, we calculated the fold changes of the metabolite concentrations between trypsinized and scraped cell samples. For visualization, metabolite concentrations of scraped samples of all cell lines were set to 1 as indicated by the horizontal red line. Identities of the single metabolites are denoted on the x axis and metabolite classes are grouped as indicated by colored bars. Fold changes of metabolite levels in trypsinized cells to those in scraped cells are denoted by dots (color coded for different cell lines).

Table S-1 List of measured metabolites with their abbreviations, names, metabolite classes, HMDB IDs, and MSI level of metabolite identification.

The table lists metabolites covered by the measured MRMs, their abbreviations, names, metabolite classes, MSI level of metabolite identification, and, if available, HMDB IDs. Based on the MSI standards (Salek et al. 2013), the levels were defined as follows: Level 1 are metabolites, which were measured with LC-MS/MS (two orthogonal properties: retention time and MRM) and which had the same isotopically labeled metabolite as internal standard. Level 2 are metabolites, which were either measured with FIA-MS/MS and/or did not have an identical isotopically labeled internal standard. Chirality was not taken into account for classification.

Abbreviation	Name	Metabolite class	HMDB ID	MSI level
C0	Carnitine	(Acyl)carnitines		Level 2
C0	D-Carnitine	(Acyl)carnitines	HMDB00062	Level 2
C0	L-Carnitine	(Acyl)carnitines		Level 2
C2	Acetylcarnitine	Acylcarnitines	HMDB00201	Level 2
C2	Acetyl-D-carnitine	Acylcarnitines		Level 2
C2	Acetyl-L-carnitine	Acylcarnitines	HMDB00201	Level 2
C3	Propionylcarnitine	Acylcarnitines	HMDB00824	Level 2
C3	Propionyl-L-carnitine	Acylcarnitines	HMDB00824	Level 2
C3-DC (C4-OH)	Malonylcarnitine	Acylcarnitines	HMDB02095	Level 2
C3-DC (C4-OH)	Malonyl-D-carnitine	Acylcarnitines	HMDB02095	Level 2
C3-DC (C4-OH)	Malonyl-L-carnitine	Acylcarnitines	HMDB02095	Level 2
C3-DC (C4-OH)	Hydroxybutyrylcarnitine	Acylcarnitines	HMDB13127	
C3-OH	Hydroxypropionylcarnitine	Acylcarnitines		Level 2
C3:1	Propenylcarnitine	Acylcarnitines		Level 2
C4	Butyrylcarnitine	Acylcarnitines	HMDB02013	Level 2
C4	Butyryl-L-carnitine	Acylcarnitines	HMDB02013	Level 2
C4	Isobutyrylcarnitine	Acylcarnitines	HMDB00736	Level 2
C4	Isobutyryl-L-carnitine	Acylcarnitines	HMDB00736	Level 2
C4:1	Butenyl-L-carnitine	Acylcarnitines	HMDB13126	Level 2
C5	2-Methylbutyrylcarnitine	Acylcarnitines	HMDB00378	Level 2
C5	Isovalerylcarnitine	Acylcarnitines	HMDB00688	Level 2
C5	Isovalery-L-carnitine	Acylcarnitines	HMDB00688	Level 2
C5	Pivaloylcarnitine	Acylcarnitines		Level 2
C5	Valerylcarnitine	Acylcarnitines	HMDB13128	Level 2
C5	Valeryl-L-carnitine	Acylcarnitines	HMDB13128	Level 2
C5-DC (C6-OH)	Glutarylcarnitine (Hydroxyhexanoylcarnitine)	Acylcarnitines		Level 2
C5-M-DC	Methylglutaryl-L-carnitine	Acylcarnitines	HMDB00552	Level 2
C5-OH (C3-DC-M)	Hydroxyvalerylcarnitine (Methylmalonylcarnitine)	Acylcarnitines		Level 2
C5:1	Tiglylcarnitine	Acylcarnitines	HMDB02366	Level 2
C5:1-DC	Glutaconylcarnitine	Acylcarnitines		Level 2

C6 (C4:1-DC)	Hexanoylcarnitine (Fumarylcarnitine)	Acylcarnitines	HMDB00705	Level 2
C6 (C4:1-DC)	L-Hexanoylcarnitine	Acylcarnitines	HMDB00756	Level 2
C6:1	Hexenoylcarnitine	Acylcarnitines		Level 2
C7-DC	Pimelylcarnitine	Acylcarnitines		Level 2
C8	Octanoylcarnitine	Acylcarnitines	HMDB00791	Level 2
C8	Octanoyl-D-carnitine	Acylcarnitines		Level 2
C8	Octanoyl-L-carnitine	Acylcarnitines	HMDB00791	Level 2
C9	2,6 Dimethylheptanoylcarnitine	Acylcarnitines	HMDB06320	Level 2
C10	Decanoylcarnitine	Acylcarnitines	HMDB00651	Level 2
C10	Decanoyl-L-carnitine	Acylcarnitines	HMDB00651	Level 2
C10:1	Decenoylcarnitine	Acylcarnitines		Level 2
C10:2	Decadienylcarnitine	Acylcarnitines		Level 2
C12	Dodecanoylcarnitine	Acylcarnitines	HMDB02250	Level 2
C12	Dodecanoyl-L-carnitine	Acylcarnitines	HMDB02250	Level 2
C12-DC	Dodecanedioylcarnitine	Acylcarnitines	HMDB13327	Level 2
C12:1	Dodecenoylcarnitine	Acylcarnitines		Level 2
C14	Myristylcarnitine	Acylcarnitines		Level 2
C14	Myristyl-L-carnitine	Acylcarnitines		Level 2
C14:1	cis-5-Tetradecenoylcarnitine	Acylcarnitines	HMDB02014	Level 2
C14:1	Tetradecenoylcarnitine	Acylcarnitines		Level 2
C14:1-OH	Hydroxytetradecenoylcarnitine	Acylcarnitines		Level 2
C14:2	Tetradecadienylcarnitine	Acylcarnitines		Level 2
C14:2-OH	Hydroxytetradecadienylcarnitine	Acylcarnitines		Level 2
C16	Palmitoylcarnitine	Acylcarnitines	HMDB00222	Level 2
C16	D-Palmitoylcarnitine	Acylcarnitines	HMDB00222	Level 2
C16	L-Palmitoylcarnitine	Acylcarnitines	HMDB00222	Level 2
C16-OH	Hydroxyhexadecanoylcarnitine	Acylcarnitines		Level 2
C16:1	trans-Hexadec-2-enoyl carnitine	Acylcarnitines	HMDB06317	Level 2
C16:1-OH	Hydroxyhexadecenoylcarnitine	Acylcarnitines		Level 2
C16:2	Hexadecadienylcarnitine	Acylcarnitines		Level 2
C16:2-OH	Hydroxyhexadecadienylcarnitine	Acylcarnitines		Level 2
C18	Stearoylcarnitine	Acylcarnitines	HMDB00848	Level 2
C18:1	Oleoylcarnitine	Acylcarnitines	HMDB06464	Level 2
C18:1	Elaidic carnitine	Acylcarnitines	HMDB06464	Level 2
C18:1-OH	Hydroxyoctadecenoylcarnitine	Acylcarnitines		Level 2
C18:2	Linoelaidylcarnitine	Acylcarnitines	HMDB06461	Level 2
Ala	Alanine	amino acids		Level 1
Ala	D-Alanine	amino acids	HMDB01310	Level 1
Ala	L-Alanine	amino acids	HMDB00161	Level 1
Ala	beta-Alanine	amino acids	HMDB00056	Level 1
Arg	Arginine	amino acids		Level 1
Arg	D-Arginine	amino acids	HMDB03416	Level 1
Arg	L-Arginine	amino acids	HMDB00517	Level 1

Asn	Asparagine	amino acids		Level 1
Asn	D-Asparagine	amino acids	HMDB33780	Level 1
Asn	L-Asparagine	amino acids	HMDB00168	Level 1
Asp	Aspartic acid	amino acids		Level 1
Asp	D-Aspartic acid	amino acids	HMDB06483	Level 1
Asp	L-Aspartic acid	amino acids	HMDB00191	Level 1
Cit	Citrulline	amino acids		Level 1
Cit	D-Citrulline	amino acids		Level 1
Cit	L-Citrulline	amino acids	HMDB00904	Level 1
Gln	Glutamine	amino acids		Level 1
Gln	D-Glutamine	amino acids	HMDB03423	Level 1
Gln	L-Glutamine	amino acids	HMDB00641	Level 1
Glu	Glutamic acid	amino acids		Level 1
Glu	D-Glutamic acid	amino acids	HMDB03339	Level 1
Glu	L-Glutamic acid	amino acids	HMDB00148	Level 1
Gly	Glycine	amino acids	HMDB00123	Level 1
His	Histidine	amino acids		Level 1
His	D-Histidine	amino acids		Level 1
His	L-Histidine	amino acids	HMDB00177	Level 1
Ile	Isoleucine	amino acids		Level 1
Ile	D-Isoleucine	amino acids		Level 1
Ile	L-Isoleucine	amino acids	HMDB00172	Level 1
Ile	Allo-D-Isoleucine	amino acids		Level 1
Ile	Allo-L-Isoleucine	amino acids	HMDB00557	Level 1
Leu	Leucine	amino acids		Level 2
Leu	D-Leucine	amino acids		Level 2
Leu	L-Leucine	amino acids	HMDB00687	Level 2
Leu	beta-Leucine	amino acids		Level 2
Leu	D-beta-Leucine	amino acids	HMDB03640	Level 2
Leu	L-beta-Leucine	amino acids	HMDB03640	Level 2
Lys	Lysine	amino acids		Level 2
Lys	D-Lysine	amino acids	HMDB03405	Level 2
Lys	L-Lysine	amino acids	HMDB00182	Level 2
Met	Methionine	amino acids		Level 1
Met	D-Methionine	amino acids		Level 1
Met	L-Methionine	amino acids	HMDB00696	Level 1
Orn	Ornithine	amino acids		Level 1
Orn	D-Ornithine	amino acids	HMDB03374	Level 1
Orn	L-Ornithine	amino acids	HMDB00214	Level 1
Phe	Phenylalanine	amino acids		Level 1
Phe	D-Phenylalanine	amino acids		Level 1
Phe	L-Phenylalanine	amino acids	HMDB00159	Level 1
Pro	Proline	amino acids		Level 1

Pro	D-Proline	amino acids	HMDB03411	Level 1
Pro	L-Proline	amino acids	HMDB00162	Level 1
Ser	Serine	amino acids		Level 1
Ser	D-Serine	amino acids	HMDB03406	Level 1
Ser	L-Serine	amino acids	HMDB00187	Level 1
Thr	Threonine	amino acids		Level 1
Thr	D-Allothreonine	amino acids		Level 1
Thr	L-Allothreonine	amino acids	HMDB04041	Level 1
Thr	L-Threonine	amino acids	HMDB00167	Level 1
Trp	Tryptophan	amino acids		Level 1
Trp	D-Tryptophan	amino acids	HMDB13609	Level 1
Trp	L-Tryptophan	amino acids	HMDB00929	Level 1
Tyr	Tyrosine	amino acids		Level 1
Tyr	D-Tyrosine	amino acids		Level 1
Tyr	L-Tyrosine	amino acids	HMDB00158	Level 1
Val	Valine	amino acids		Level 1
Val	D-Valine	amino acids		Level 1
Val	L-Valine	amino acids	HMDB00883	Level 1
ADMA	Asymmetric dimethylarginine	biogenic amines	HMDB01539	Level 1
Ac-Orn	Acetylornithine	biogenic amines	HMDB03357	Level 2
Carnosine	Carnosine	biogenic amines		Level 2
Carnosine	L-Carnosine	biogenic amines	HMDB00033	Level 2
Creatinine	Creatinine	biogenic amines	HMDB00562	Level 1
DOPA	Dihydroxyphenylalanine	biogenic amines	HMDB00609	Level 1
DOPA	Dihydroxyphenyl-D-alanine	biogenic amines		Level 1
DOPA	L-Dihydroxyphenylalanine	biogenic amines	HMDB00181	Level 1
Dopamine	Dopamine	biogenic amines	HMDB00073	Level 1
Histamine	Histamine	biogenic amines	HMDB00870	Level 2
Kynurenine	Kynurenine	biogenic amines		Level 2
Kynurenine	D-Kynurenine	biogenic amines		Level 2
Kynurenine	L-Kynurenine	biogenic amines	HMDB00684	Level 2
Met-SO	Methionine-Sulfoxide	biogenic amines		Level 2
Met-SO	L-Methionine sulfoxide	biogenic amines	HMDB02005	Level 2
Met-SO	L-Methionine-(R)-Sulfoxide	biogenic amines		Level 2
Met-SO	L-Methionine-(S)-Sulfoxide	biogenic amines		Level 2
Nitro-Tyr	2-Nitrotyrosine	biogenic amines		Level 2
Nitro-Tyr	3-Nitrotyrosine	biogenic amines	HMDB01904	Level 2
Nitro-Tyr	Nitro-D-tyrosine	biogenic amines		Level 2
Nitro-Tyr	Nitro-L-tyrosine	biogenic amines		Level 2
OH-Pro	4-Hydroxyproline	biogenic amines	HMDB00725	Level 2
OH-Pro	3-Hydroxy-L-proline	biogenic amines	HMDB02113	Level 2
OH-Pro	4-Hydroxy-L-proline	biogenic amines	HMDB00725	Level 2
OH-Pro	cis-4-Hydroxy-L-proline	biogenic amines	HMDB06055	Level 2

Metabolomics

PEA	(1S)-Phenylethylamine	biogenic amines		Level 2
PEA	1-Phenylethylamine	biogenic amines	HMDB02017	Level 2
PEA	2-Phenylethylamine	biogenic amines	HMDB12275	Level 2
Putrescine	Putrescine	biogenic amines	HMDB01414	Level 1
SDMA	Symmetric dimethylarginine	biogenic amines	HMDB03334	Level 2
SDMA	L-Symmetric dimethylarginine	biogenic amines		Level 2
Serotonin	Serotonin	biogenic amines	HMDB00259	Level 1
Spermidine	Spermidine	biogenic amines	HMDB01257	Level 1
Spermine	Spermine	biogenic amines	HMDB01256	Level 1
Taurine	Taurine	biogenic amines	HMDB00251	Level 1
alpha-AAA	2-Aminoadipic acid	biogenic amines		Level 2
alpha-AAA	D-2-Aminoadipic acid	biogenic amines		Level 2
alpha-AAA	L-2-Aminoadipic acid	biogenic amines	HMDB00510	Level 2
lysoPC a C14:0	C14:0 a Lyso-phosphatidylcholine	Lyso-phosphatidylcholines		Level 2
lysoPC a C14:0	LysoPC(14:0)	Lyso-phosphatidylcholines	HMDB10379	Level 2
lysoPC a C14:0	C14:0 2a L-Lyso-phosphatidylcholine	Lyso-phosphatidylcholines		Level 2
lysoPC a C16:0	C16:0 a Lyso-phosphatidylcholine	Lyso-phosphatidylcholines		Level 2
lysoPC a C16:0	C16:0 a D-Lyso-phosphatidylcholine	Lyso-phosphatidylcholines		Level 2
lysoPC a C16:0	LysoPC(16:0)	Lyso-phosphatidylcholines	HMDB10382	Level 2
lysoPC a C16:0	C16:0 2a Lyso-phosphatidylcholine	Lyso-phosphatidylcholines		Level 2
lysoPC a C16:0	C16:0 2a L-Lyso-phosphatidylcholine	Lyso-phosphatidylcholines		Level 2
lysoPC a C16:1	C16:1;9Z-enoyl a Lyso-phosphatidylcholine	Lyso-phosphatidylcholines		Level 2
lysoPC a C16:1	C16:1,9E-enoyl a L-Lyso-phosphatidylcholine	Lyso-phosphatidylcholines		Level 2
lysoPC a C16:1	LysoPC(16:1(9Z))	Lyso-phosphatidylcholines	HMDB10383	Level 2
lysoPC a C17:0	C17:0 a Lyso-phosphatidylcholine	Lyso-phosphatidylcholines		Level 2
lysoPC a C17:0	LysoPC(17:0)	Lyso-phosphatidylcholines	HMDB12108	Level 2
lysoPC a C18:0	C18:0 a Lyso-phosphatidylcholine	Lyso-phosphatidylcholines		Level 2
lysoPC a C18:0	C18:0 a D-Lyso-phosphatidylcholine	Lyso-phosphatidylcholines		Level 2
lysoPC a C18:0	LysoPC(18:0)	Lyso-phosphatidylcholines	HMDB10384	Level 2
lysoPC a C18:0	C18:0 2a Lyso-phosphatidylcholine	Lyso-phosphatidylcholines		Level 2
lysoPC a C18:0	C18:0 2a D-Lyso-phosphatidylcholine	Lyso-phosphatidylcholines		Level 2
lysoPC a C18:0	LysoPC(0:0/18:0)	Lyso-phosphatidylcholines	HMDB11128	Level 2
lysoPC a C18:1	C18:1;9E-enoyl a Lyso-phosphatidylcholine	Lyso-phosphatidylcholines		Level 2
lysoPC a C18:1	C18:1;9E-enoyl a L-Lyso-phosphatidylcholine	Lyso-phosphatidylcholines		Level 2
lysoPC a C18:1	C18:1;9E-enoyl 2a Lyso-phosphatidylcholine	Lyso-		Level 2

		phosphatidylcholines		
lysoPC a C18:1	C18:1;9E-enoyl 2a L-Lyso-phosphatidylcholine	Lyso-phosphatidylcholines		Level 2
lysoPC a C18:1	C18:1;6Z-enoyl a L-Lyso-phosphatidylcholine	Lyso-phosphatidylcholines		Level 2
lysoPC a C18:1	C18:1;6Z-enoyl 2a L-Lyso-phosphatidylcholine	Lyso-phosphatidylcholines		Level 2
lysoPC a C18:1	C18:1;9Z-enoyl a Lyso-phosphatidylcholine	Lyso-phosphatidylcholines		Level 2
lysoPC a C18:1	LysoPC(18:1(9Z))	Lyso-phosphatidylcholines	HMDB02815	Level 2
lysoPC a C18:1	C18:1;9Z-enoyl 2a Lyso-phosphatidylcholine	Lyso-phosphatidylcholines		Level 2
lysoPC a C18:1	C18:1;9Z-enoyl 2a L-Lyso-phosphatidylcholine	Lyso-phosphatidylcholines		Level 2
lysoPC a C18:1	LysoPC(18:1(11Z))	Lyso-phosphatidylcholines	HMDB10385	Level 2
lysoPC a C18:2	C18:1;9Z,12Z-dienoyl a Lyso-phosphatidylcholine	Lyso-phosphatidylcholines		Level 2
lysoPC a C18:2	C18:1;2E,4E-dienoyl a L-Lyso-phosphatidylcholine	Lyso-phosphatidylcholines		Level 2
lysoPC a C18:2	LysoPC(18:2(9Z,12Z))	Lyso-phosphatidylcholines	HMDB10386	Level 2
lysoPC a C20:3	lysoPC a C20:3	Lyso-phosphatidylcholines		Level 2
lysoPC a C20:3	LysoPC(20:3(5Z,8Z,11Z))	Lyso-phosphatidylcholines	HMDB10393	Level 2
lysoPC a C20:3	LysoPC(20:3(8Z,11Z,14Z))	Lyso-phosphatidylcholines	HMDB10394	Level 2
lysoPC a C20:4	(5Z,8Z,11Z,14Z) a C20:4 Lyso-phosphatidylcholine	Lyso-phosphatidylcholines		Level 2
lysoPC a C20:4	LysoPC(20:4(5Z,8Z,11Z,14Z))	Lyso-phosphatidylcholines	HMDB10395	Level 2
lysoPC a C20:4	LysoPC(20:4(8Z,11Z,14Z,17Z))	Lyso-phosphatidylcholines	HMDB10396	Level 2
lysoPC a C24:0	C24:0 a Lyso-phosphatidylcholine	Lyso-phosphatidylcholines		Level 2
lysoPC a C24:0	LysoPC(24:0)	Lyso-phosphatidylcholines	HMDB10405	Level 2
lysoPC a C26:0	Lyso-phosphatidylcholine a C26:0	Lyso-phosphatidylcholines		Level 2
lysoPC a C26:1	Lyso-phosphatidylcholine a C26:1	Lyso-phosphatidylcholines		Level 2
lysoPC a C28:0	Lyso-phosphatidylcholine a C28:0	Lyso-phosphatidylcholines		Level 2
lysoPC a C28:1	Lyso-phosphatidylcholine a C28:1	Lyso-phosphatidylcholines		Level 2
PC aa C26:0	C(10:0/16:0) aa Phosphatidylcholine	Phosphatidylcholines		Level 2
PC aa C26:0	C(11:0/15:0) aa Phosphatidylcholine	Phosphatidylcholines		Level 2
PC aa C26:0	C(12:0/14:0) aa L-Phosphatidylcholine	Phosphatidylcholines		Level 2
PC aa C26:0	C(13:0/13:0) aa Phosphatidylcholine	Phosphatidylcholines		Level 2
PC aa C26:0	C(13:0/13:0) aa L- Phosphatidylcholine	Phosphatidylcholines		Level 2
PC aa C26:0	C(14:0/12:0) aa Phosphatidylcholine	Phosphatidylcholines		Level 2
PC aa C26:0	C(14:0/12:0) aa L-Phosphatidylcholine	Phosphatidylcholines		Level 2
PC aa C26:0	C(18:0/8:0) aa L-Phosphatidylcholine	Phosphatidylcholines		Level 2
PC aa C26:0	C(5:0/11:0) aa Phosphatidylcholine	Phosphatidylcholines		Level 2
PC aa C26:0	C(8:0/18:0) aa Phosphatidylcholine	Phosphatidylcholines		Level 2

Metabolomics

PC aa C26:0	C(9:0/17:0) aa Phosphatidylcholine	Phosphatidylcholines		Level 2
PC aa C26:0	C(9:0/17:0) aa L-Phosphatidylcholine	Phosphatidylcholines		Level 2
PC aa C28:1	C(10:0/18:1;9Z-enoyl) aa L-Phosphatidylcholine	Phosphatidylcholines		Level 2
PC aa C30:0	C(12:0/18:0) aa Phosphatidylcholine	Phosphatidylcholines		Level 2
PC aa C30:0	C(12:0/18:0) aa L-Phosphatidylcholine	Phosphatidylcholines		Level 2
PC aa C30:0	C(10:0/20:0) aa L-Phosphatidylcholine	Phosphatidylcholines		Level 2
PC aa C30:0	C(11:0/19:0) aa L-Phosphatidylcholine	Phosphatidylcholines		Level 2
PC aa C30:0	C(13:0/17:0) aa L-Phosphatidylcholine	Phosphatidylcholines		Level 2
PC aa C30:0	C(14:0/16:0) aa Phosphatidylcholine	Phosphatidylcholines		Level 2
PC aa C30:0	PC(16:0/14:0)	Phosphatidylcholines	HMDB07965	Level 2
PC aa C30:0	C(15:0/15:0) aa Phosphatidylcholine	Phosphatidylcholines		Level 2
PC aa C30:0	PC(15:0/15:0)	Phosphatidylcholines	HMDB07934	Level 2
PC aa C30:0	C(16:0/14:0) aa Phosphatidylcholine	Phosphatidylcholines		Level 2
PC aa C30:0	C(16:0/14:0) aa D-Phosphatidylcholine	Phosphatidylcholines		Level 2
PC aa C30:0	PC(14:0/16:0)	Phosphatidylcholines	HMDB07869	Level 2
PC aa C30:0	C(17:0/13:0) aa L-Phosphatidylcholine	Phosphatidylcholines		Level 2
PC aa C30:0	C(18:0/12:0) aa Phosphatidylcholine	Phosphatidylcholines		Level 2
PC aa C30:0	C(18:0/12:0) aa L-Phosphatidylcholine	Phosphatidylcholines		Level 2
PC aa C30:0	C(19:0/11:0) aa L-Phosphatidylcholine	Phosphatidylcholines		Level 2
PC aa C30:0	C(20:0/10:0) aa L-Phosphatidylcholine	Phosphatidylcholines		Level 2
PC aa C30:0	C(21:0/9:0) aa L-Phosphatidylcholine	Phosphatidylcholines		Level 2
PC aa C30:2	Phosphatidylcholine with diacyl residue sum C30:2	Phosphatidylcholines		Level 2
PC aa C32:0	C(18:0/14:0) aa Phosphatidylcholine	Phosphatidylcholines		Level 2
PC aa C32:0	PC(14:0/18:0)	Phosphatidylcholines	HMDB07871	Level 2
PC aa C32:0	C(10:0/22:0) aa L-Phosphatidylcholine	Phosphatidylcholines		Level 2
PC aa C32:0	C(11:0/21:0) aa L-Phosphatidylcholine	Phosphatidylcholines		Level 2
PC aa C32:0	C(12:0/20:0) aa Phosphatidylcholine	Phosphatidylcholines		Level 2
PC aa C32:0	C(12:0/20:0) aa L-Phosphatidylcholine	Phosphatidylcholines		Level 2
PC aa C32:0	C(13:0/19:0) aa L-Phosphatidylcholine	Phosphatidylcholines		Level 2
PC aa C32:0	C(14:0/18:0) aa Phosphatidylcholine	Phosphatidylcholines		Level 2
PC aa C32:0	PC(18:0/14:0)	Phosphatidylcholines	HMDB08031	Level 2
PC aa C32:0	C(15:0/17:0) aa Phosphatidylcholine	Phosphatidylcholines		Level 2
PC aa C32:0	C(15:0/17:0) aa L-Phosphatidylcholine	Phosphatidylcholines		Level 2
PC aa C32:0	C(16:0/16:0) aa Phosphatidylcholine	Phosphatidylcholines		Level 2
PC aa C32:0	PC(16:0/16:0)	Phosphatidylcholines	HMDB00564	Level 2
PC aa C32:0	C(17:0/15:0) aa Phosphatidylcholine	Phosphatidylcholines		Level 2
PC aa C32:0	C(17:0/15:0) aa L-Phosphatidylcholine	Phosphatidylcholines		Level 2
PC aa C32:0	C(19:0/13:0) aa Phosphatidylcholine	Phosphatidylcholines		Level 2
PC aa C32:0	C(19:0/13:0) aa L-Phosphatidylcholine	Phosphatidylcholines		Level 2
PC aa C32:0	C(20:0/12:0) aa Phosphatidylcholine	Phosphatidylcholines		Level 2
PC aa C32:0	C(20:0/12:0) aa L-Phosphatidylcholine	Phosphatidylcholines		Level 2
PC aa C32:0	C(21:0/11:0) aa L-Phosphatidylcholine	Phosphatidylcholines		Level 2

PC aa C32:0	C(22:0/10:0) aa Phosphatidylcholine	Phosphatidylcholines		Level 2
PC aa C32:0	C(22:0/10:0) aa L-Phosphatidylcholine	Phosphatidylcholines		Level 2
PC aa C32:0	C(23:0/9:0) aa L-Phosphatidylcholine	Phosphatidylcholines		Level 2
PC aa C32:1	C(18:1;9E-enoyl/14:0) aa Phosphatidylcholine	Phosphatidylcholines		Level 2
PC aa C32:1	PC(14:0/18:1(11Z))	Phosphatidylcholines	HMDB07872	Level 2
PC aa C32:1	C(14:0/18:1;9Z-enoyl)) aa Phosphatidylcholine	Phosphatidylcholines		Level 2
PC aa C32:1	PC(18:1(9Z)/14:0)	Phosphatidylcholines	HMDB08097	Level 2
PC aa C32:1	C(16:1;9Z-enoyl)/16:0) aa Phosphatidylcholine	Phosphatidylcholines		Level 2
PC aa C32:1	PC(16:0/16:1(9Z))	Phosphatidylcholines	HMDB07969	Level 2
PC aa C32:1	C(18:1;9Z-enoyl)/14:0) aa Phosphatidylcholine	Phosphatidylcholines		Level 2
PC aa C32:1	PC(14:0/18:1(9Z))	Phosphatidylcholines	HMDB07873	Level 2
PC aa C32:2	C(16:1;9E-enoyl/16:1;9E-enoyl) aa Phosphatidylcholine	Phosphatidylcholines		Level 2
PC aa C32:2	C(16:1;9E-enoyl/16:1;9E-enoyl) aa L-Phosphatidylcholine	Phosphatidylcholines		Level 2
PC aa C32:2	C(16:1;9Z-enoyl/16:1;9Z-enoyl) aa Phosphatidylcholine	Phosphatidylcholines		Level 2
PC aa C32:2	PC(16:1(9Z)/16:1(9Z))	Phosphatidylcholines	HMDB08002	Level 2
PC aa C32:2	C(18:2;2E,4E-dienoyl/14:0) aa Phosphatidylcholine	Phosphatidylcholines		Level 2
PC aa C32:2	C(18:2;11Z,14Z-dienoyl/14:0) aa L-Phosphatidylcholine	Phosphatidylcholines		Level 2
PC aa C32:2	PC(14:0/18:2(9Z,12Z))	Phosphatidylcholines	HMDB07874	Level 2
PC aa C32:3	C(18:3;9Z,12Z,15Z-trienoyl/14:0) aa Phosphatidylcholine	Phosphatidylcholines		Level 2
PC aa C32:3	PC(14:0/18:3(9Z,12Z,15Z))	Phosphatidylcholines	HMDB07876	Level 2
PC aa C34:1	PC(18:1(9Z)/16:0)	Phosphatidylcholines	HMDB08100	Level 2
PC aa C34:1	C(16:0/18:1;9Z-enoyl) aa L-Phosphatidylcholine	Phosphatidylcholines		Level 2
PC aa C34:1	C(16:0/18:1;9Z-enoyl) aa Phosphatidylcholine	Phosphatidylcholines		Level 2
PC aa C34:1	PC(18:0/16:1(9Z))	Phosphatidylcholines	HMDB08035	Level 2
PC aa C34:1	C(18:0/16:1;7Z-enoyl) aa Phosphatidylcholine	Phosphatidylcholines		Level 2
PC aa C34:1	PC(16:1(9Z)/18:0)	Phosphatidylcholines	HMDB08003	Level 2
PC aa C34:1	C(18:1;11E-enoyl/16:0) aa L-Phosphatidylcholine	Phosphatidylcholines		Level 2
PC aa C34:1	C(18:1;11Z-enoyl/16:0) aa Phosphatidylcholine	Phosphatidylcholines		Level 2
PC aa C34:1	PC(16:0/18:1(11Z))	Phosphatidylcholines	HMDB07971	Level 2
PC aa C34:1	C(18:1;6Z-enoyl/16:0) aa Phosphatidylcholine	Phosphatidylcholines		Level 2
PC aa C34:1	C(18:1;6E-enoyl/16:0) aa L-Phosphatidylcholine	Phosphatidylcholines		Level 2
PC aa C34:1	C(18:1;6Z-enoyl/16:0)) aa L-Phosphatidylcholine	Phosphatidylcholines		Level 2
PC aa C34:1	PC(16:0/18:1(9Z))	Phosphatidylcholines	HMDB07972	Level 2
PC aa C34:1	C(18:1;9E-enoyl/16:0)) aa D-Phosphatidylcholine	Phosphatidylcholines		Level 2
PC aa C34:1	C(18:1;9E-enoyl/16:0)) aa L-Phosphatidylcholine	Phosphatidylcholines		Level 2
PC aa C34:1	C(18:1;9Z-enoyl/16:0)) aa Phosphatidylcholine	Phosphatidylcholines		Level 2
PC aa C34:1	C(18:1;9Z-enoyl/16:0) aa L-Phosphatidylcholine	Phosphatidylcholines		Level 2
PC aa C34:2	C(16:0/18:2;2E,4E-dienoyl) aa L-	Phosphatidylcholines		Level 2

	Phosphatidylcholine			
PC aa C34:2	C(16:0/18:2;6Z,9Z-dienoyl) aa L-Phosphatidylcholine	Phosphatidylcholines		Level 2
PC aa C34:2	C(16:0/18:2;9Z,12Z-dienoyl) aa Phosphatidylcholine	Phosphatidylcholines		Level 2
PC aa C34:2	PC(18:2(9Z,12Z)/16:0)	Phosphatidylcholines	HMDB08133	Level 2
PC aa C34:2	C(16:1;2E,4E-dienoyl/18:0) aa L-Phosphatidylcholine	Phosphatidylcholines		Level 2
PC aa C34:2	C(16:1;3E-enoyl/18:1;9Z-enoyl) aa Phosphatidylcholine	Phosphatidylcholines		Level 2
PC aa C34:2	PC(18:1(9Z)/16:1(9Z))	Phosphatidylcholines	HMDB08101	Level 2
PC aa C34:2	C(17:1;10E-enoyl/17:1;10E-enoyl) aa Phosphatidylcholine	Phosphatidylcholines		Level 2
PC aa C34:2	C(17:1;10E-enoyl/17:1;10Z-enoyl) aa L-Phosphatidylcholine	Phosphatidylcholines		Level 2
PC aa C34:2	C(17:1;10E-enoyl/17:1;9Z-enoyl) aa L-Phosphatidylcholine	Phosphatidylcholines		Level 2
PC aa C34:2	PC(16:1(9Z)/18:1(11Z))	Phosphatidylcholines	HMDB08004	Level 2
PC aa C34:2	C(18:1;9Z-enoyl/16:1;2Z-enoyl) aa L-Phosphatidylcholine	Phosphatidylcholines		Level 2
PC aa C34:2	C(18:1;9Z-enoyl/16:1;7Z-enoyl) aa Phosphatidylcholine	Phosphatidylcholines		Level 2
PC aa C34:2	C(18:1;9Z-enoyl/16:1;9Z-enoyl) aa Phosphatidylcholine	Phosphatidylcholines		Level 2
PC aa C34:2	PC(16:1(9Z)/18:1(9Z))	Phosphatidylcholines	HMDB08005	Level 2
PC aa C34:2	C(18:2;10E,12Z-dienoyl/16:0) aa L-Phosphatidylcholine	Phosphatidylcholines		Level 2
PC aa C34:2	C(18:2;11Z,13Z-dienoyl/16:0) aa L-Phosphatidylcholine	Phosphatidylcholines		Level 2
PC aa C34:2	C(18:2;2E,4E-dienoyl/16:0) aa L-Phosphatidylcholine	Phosphatidylcholines		Level 2
PC aa C34:2	C(18:2;2Z,4Z-dienoyl/16:0) aa L-Phosphatidylcholine	Phosphatidylcholines		Level 2
PC aa C34:2	C(18:2;6Z,9Z-dienoyl/16:0) aa L-Phosphatidylcholine	Phosphatidylcholines		Level 2
PC aa C34:2	C(18:2;9E,11E-dienoyl/16:0) aa L-Phosphatidylcholine	Phosphatidylcholines		Level 2
PC aa C34:2	C(18:2;9E,11Z-dienoyl/16:0) aa L-Phosphatidylcholine	Phosphatidylcholines		Level 2
PC aa C34:2	PC(16:0/18:2(9Z,12Z))	Phosphatidylcholines	HMDB07973	Level 2
PC aa C34:2	C(18:2;9E,12E-dienoyl/16:0) aa L-Phosphatidylcholine	Phosphatidylcholines		Level 2
PC aa C34:2	C(18:2;9Z,12Z-dienoyl/16:0) aa Phosphatidylcholine	Phosphatidylcholines		Level 2
PC aa C34:2	C(18:2;9Z,12Z-dienoyl/16:0) aa D-Phosphatidylcholine	Phosphatidylcholines		Level 2
PC aa C34:2	C(18:2;9Z,12Z-dienoyl/16:0) aa L-Phosphatidylcholine	Phosphatidylcholines		Level 2
PC aa C34:3	C(18:2;9Z,12Z-dienoyl/16:1;7Z-enoyl) aa Phosphatidylcholine	Phosphatidylcholines		Level 2
PC aa C34:3	C(18:2;9Z,12Z-dienoyl/16:1;9Z-enoyl) aa Phosphatidylcholine	Phosphatidylcholines		Level 2
PC aa C34:3	PC(16:1(9Z)/18:2(9Z,12Z))	Phosphatidylcholines	HMDB08006	Level 2
PC aa C34:3	C(18:3;5E,9Z,12Z-trienoyl/16:0) aa Phosphatidylcholine	Phosphatidylcholines		Level 2
PC aa C34:3	C(18:3;6Z,9Z,12Z-trienoyl/16:0) aa Phosphatidylcholine	Phosphatidylcholines		Level 2
PC aa C34:3	PC(16:0/18:3(6Z,9Z,12Z))	Phosphatidylcholines	HMDB07974	Level 2

PC aa C34:3	C(18:3;9E,12E,15E-trienoyl/16:0) aa Phosphatidylcholine	Phosphatidylcholines		Level 2
PC aa C34:3	PC(16:0/18:3(9Z,12Z,15Z))	Phosphatidylcholines	HMDB07975	Level 2
PC aa C34:3	C(18:3;9Z,12Z,15Z-trienoyl/16:0) aa Phosphatidylcholine	Phosphatidylcholines		Level 2
PC aa C34:3	C(19:3;9Z,12Z,15Z-trienoyl/15:0) aa Phosphatidylcholine	Phosphatidylcholines		Level 2
PC aa C34:4	C(16:0/18:4;9Z,11E,13E,15Z-tetraenoyl) aa L-Phosphatidylcholine	Phosphatidylcholines		Level 2
PC aa C34:4	C(16:2;5Z,8Z-dienoyl/18:2;9Z,12Z-dienoyl) aa Phosphatidylcholine	Phosphatidylcholines		Level 2
PC aa C34:4	C(17:2;9Z,11Z-dienoyl/17:2;9Z,11Z-dienoyll) aa L-Phosphatidylcholine	Phosphatidylcholines		Level 2
PC aa C34:4	C(18:2;2E,4E-dienoyl/16:2;2E,4E-dienoyl) aa L-Phosphatidylcholine	Phosphatidylcholines		Level 2
PC aa C34:4	PC(16:0/18:4(6Z,9Z,12Z,15Z))	Phosphatidylcholines	HMDB07976	Level 2
PC aa C34:4	C(18:4;9E,11E,13E,15E-tetraenoyl/16:0) aa L-Phosphatidylcholine	Phosphatidylcholines		Level 2
PC aa C34:4	C(18:4;9E,11E,13E,15E-tetraenoyl/16:0) aa Phosphatidylcholine	Phosphatidylcholines		Level 2
PC aa C34:4	C(18:4;9Z,11E,13E,15Z-tetraenoyl/16:0) aa L-Phosphatidylcholine	Phosphatidylcholines		Level 2
PC aa C34:4	C(18:4;9Z,11E,13E,15Z-tetraenoyl/16:0) aa Phosphatidylcholine	Phosphatidylcholines		Level 2
PC aa C34:4	C(18:4;9Z,11Z,13Z,15Z-tetraenoyl/16:0) aa L-Phosphatidylcholine	Phosphatidylcholines		Level 2
PC aa C34:4	PC(14:0/20:4(5Z,8Z,11Z,14Z))	Phosphatidylcholines	HMDB07883	Level 2
PC aa C34:4	C(20:4;5Z,8Z,11Z,14Z-tetraenoyl/14:0) aa Phosphatidylcholine	Phosphatidylcholines		Level 2
PC aa C36:0	C(11:0/25:0) aa Phosphatidylcholine	Phosphatidylcholines		Level 2
PC aa C36:0	C(12:0/24:0) aa Phosphatidylcholine	Phosphatidylcholines		Level 2
PC aa C36:0	C(13:0/23:0) aa Phosphatidylcholine	Phosphatidylcholines		Level 2
PC aa C36:0	PC(22:0/14:0)	Phosphatidylcholines	HMDB08525	Level 2
PC aa C36:0	C(15:0/21:0) aa Phosphatidylcholine	Phosphatidylcholines		Level 2
PC aa C36:0	PC(20:0/16:0)	Phosphatidylcholines	HMDB08265	Level 2
PC aa C36:0	C(17:0/19:0) aa Phosphatidylcholine	Phosphatidylcholines		Level 2
PC aa C36:0	PC(18:0/18:0)	Phosphatidylcholines	HMDB08036	Level 2
PC aa C36:0	C(18:0/18:0) aa L-Phosphatidylcholine	Phosphatidylcholines		Level 2
PC aa C36:0	C(18:0/18:0) aa Phosphatidylcholine	Phosphatidylcholines		Level 2
PC aa C36:0	C(19:0/17:0) aa Phosphatidylcholine	Phosphatidylcholines		Level 2
PC aa C36:0	PC(16:0/20:0)	Phosphatidylcholines	HMDB07977	Level 2
PC aa C36:0	C(20:0/16:0) aa Phosphatidylcholine	Phosphatidylcholines		Level 2
PC aa C36:0	C(21:0/15:0) aa L-Phosphatidylcholine	Phosphatidylcholines		Level 2
PC aa C36:0	PC(14:0/22:0)	Phosphatidylcholines	HMDB07886	Level 2
PC aa C36:0	C(23:0/13:0) aa L-Phosphatidylcholine	Phosphatidylcholines		Level 2
PC aa C36:0	C(24:0/12:0) aa L-Phosphatidylcholine	Phosphatidylcholines		Level 2
PC aa C36:0	C(24:0/12:0) aa Phosphatidylcholine	Phosphatidylcholines		Level 2
PC aa C36:0	C(25:0/11:0) aa L-Phosphatidylcholine	Phosphatidylcholines		Level 2
PC aa C36:0	C(26:0/10:0) aa Phosphatidylcholine	Phosphatidylcholines		Level 2
PC aa C36:1	C(16:0/20:1;11Z-enoyl) aa Phosphatidylcholine	Phosphatidylcholines		Level 2
PC aa C36:1	C(18:0/18:1;9E-enoyl) aa Phosphatidylcholine	Phosphatidylcholines		Level 2

PC aa C36:1	PC(18:1(9Z)/18:0)	Phosphatidylcholines	HMDB08102	Level 2
PC aa C36:1	C(18:0/18:1;9Z-enoyl) aa Phosphatidylcholine	Phosphatidylcholines		Level 2
PC aa C36:1	PC(18:0/18:1(11Z))	Phosphatidylcholines	HMDB08037	Level 2
PC aa C36:1	PC(18:1(11Z)/18:0)	Phosphatidylcholines	HMDB08069	Level 2
PC aa C36:1	C(18:1;11Z-enoyl/18:0) aa Phosphatidylcholine	Phosphatidylcholines		Level 2
PC aa C36:1	C(18:1;12Z-enoyl/18:0) aa L-Phosphatidylcholine	Phosphatidylcholines		Level 2
PC aa C36:1	C(18:1;12Z-enoyl/18:0) aa Phosphatidylcholine	Phosphatidylcholines		Level 2
PC aa C36:1	C(18:1;13Z-enoyl/18:0) aa L-Phosphatidylcholine	Phosphatidylcholines		Level 2
PC aa C36:1	C(18:1;16Z-enoyl/18:0) aa L-Phosphatidylcholine	Phosphatidylcholines		Level 2
PC aa C36:1	C(18:1;16Z-enoyl/18:0) aa Phosphatidylcholine	Phosphatidylcholines		Level 2
PC aa C36:1	C(18:1;6Z-enoyl/18:0) aa L-Phosphatidylcholine	Phosphatidylcholines		Level 2
PC aa C36:1	C(18:1;6Z-enoyl/18:0) aa Phosphatidylcholine	Phosphatidylcholines		Level 2
PC aa C36:1	C(18:1;7Z-enoyl/18:0) aa L-Phosphatidylcholine	Phosphatidylcholines		Level 2
PC aa C36:1	C(18:1;9E-enoyl/18:0) aa L-Phosphatidylcholine	Phosphatidylcholines		Level 2
PC aa C36:1	C(18:1;9E-enoyl/18:0) aa Phosphatidylcholine	Phosphatidylcholines		Level 2
PC aa C36:1	C(18:1;9Z-enoyl/18:0) aa D-Phosphatidylcholine	Phosphatidylcholines		Level 2
PC aa C36:1	PC(18:0/18:1(9Z))	Phosphatidylcholines	HMDB08038	Level 2
PC aa C36:1	C(18:1;9Z-enoyl/18:0) aa Phosphatidylcholine	Phosphatidylcholines		Level 2
PC aa C36:1	C(20:1;11Z-enoyl/16:0) aa Phosphatidylcholine	Phosphatidylcholines		Level 2
PC aa C36:2	PC(18:2(9Z,12Z)/18:0)	Phosphatidylcholines	HMDB08135	Level 2
PC aa C36:2	C(18:0/18:2;9Z,12Z-dienoyl) aa Phosphatidylcholine	Phosphatidylcholines		Level 2
PC aa C36:2	C(18:1;10E-enoyl/18:1;10E-enoyl) aa Phosphatidylcholine	Phosphatidylcholines		Level 2
PC aa C36:2	C(18:1;10Z-enoyl/18:1;10Z-enoyl) aa L-Phosphatidylcholine	Phosphatidylcholines		Level 2
PC aa C36:2	C(18:1;11E-enoyl/18:1;11E-enoyl) aa L-Phosphatidylcholine	Phosphatidylcholines		Level 2
PC aa C36:2	C(18:1;11E-enoyl/18:1;11E-enoyl) aa Phosphatidylcholine	Phosphatidylcholines		Level 2
PC aa C36:2	PC(18:1(11Z)/18:1(11Z))	Phosphatidylcholines	HMDB08070	Level 2
PC aa C36:2	C(18:1;11Z-enoyl/18:1;11Z-enoyl) aa Phosphatidylcholine	Phosphatidylcholines		Level 2
PC aa C36:2	C(18:1;12E-enoyl/18:1;12E-enoyl) aa Phosphatidylcholine	Phosphatidylcholines		Level 2
PC aa C36:2	C(18:1;12Z-enoyl/18:1;12Z-enoyl) aa L-Phosphatidylcholine	Phosphatidylcholines		Level 2
PC aa C36:2	C(18:1;13E-enoyl/18:1;13E-enoyl) aa Phosphatidylcholine	Phosphatidylcholines		Level 2
PC aa C36:2	C(18:1;13Z-enoyl/18:1;13E-enoyl) aa L-Phosphatidylcholine	Phosphatidylcholines		Level 2
PC aa C36:2	C(18:1;14E-enoyl/18:1;14E-enoyl) aa Phosphatidylcholine	Phosphatidylcholines		Level 2
PC aa C36:2	C(18:1;14Z-enoyl/18:1;14Z-enoyl) aa L-Phosphatidylcholine	Phosphatidylcholines		Level 2
PC aa C36:2	C(18:1;15E-enoyl/18:1;15E-enoyl) aa Phosphatidylcholine	Phosphatidylcholines		Level 2
PC aa C36:2	C(18:1;15Z-enoyl/18:1;15Z-enoyl) aa L-Phosphatidylcholine	Phosphatidylcholines		Level 2

PC aa C36:2	C(18:1;16E-enoyl/18:1;16E-enoyl) aa Phosphatidylcholine	Phosphatidylcholines		Level 2
PC aa C36:2	C(18:1;16Z-enoyl/18:1;16Z-enoyl) aa L-Phosphatidylcholine	Phosphatidylcholines		Level 2
PC aa C36:2	C(18:1;17-enoyl/18:1;17-enoyl) aa L-Phosphatidylcholine	Phosphatidylcholines		Level 2
PC aa C36:2	C(18:1;2E-enoyl/18:1;2E-enoyl) aa Phosphatidylcholine	Phosphatidylcholines		Level 2
PC aa C36:2	C(18:1;2Z-enoyl/18:1;2Z-enoyl) aa L-Phosphatidylcholine	Phosphatidylcholines		Level 2
PC aa C36:2	C(18:1;3E-enoyl/18:1;3E-enoyl) aa Phosphatidylcholine	Phosphatidylcholines		Level 2
PC aa C36:2	C(18:1;3Z-enoyl/18:1;3Z-enoyl) aa L-Phosphatidylcholine	Phosphatidylcholines		Level 2
PC aa C36:2	C(18:1;4E-enoyl/18:1;4E-enoyl) aa Phosphatidylcholine	Phosphatidylcholines		Level 2
PC aa C36:2	C(18:1;4Z-enoyl/18:1;4Z-enoyl) aa Phosphatidylcholine	Phosphatidylcholines		Level 2
PC aa C36:2	C(18:1;5E-enoyl/18:1;5E-enoyl) aa Phosphatidylcholine	Phosphatidylcholines		Level 2
PC aa C36:2	C(18:1;5Z-enoyl/18:1;5Z-enoyl) aa L-Phosphatidylcholine	Phosphatidylcholines		Level 2
PC aa C36:2	C(18:1;6E-enoyl/18:1;6E-enoyl) aa L-Phosphatidylcholine	Phosphatidylcholines		Level 2
PC aa C36:2	C(18:1;6E-enoyl/18:1;6E-enoyl) aa Phosphatidylcholine	Phosphatidylcholines		Level 2
PC aa C36:2	C(18:1;6Z-enoyl/18:1;6Z-enoyl) aa L-Phosphatidylcholine	Phosphatidylcholines		Level 2
PC aa C36:2	C(18:1;6Z-enoyl/18:1;6Z-enoyl) aa Phosphatidylcholine	Phosphatidylcholines		Level 2
PC aa C36:2	C(18:1;7E-enoyl/18:1;7E-enoyl) aa Phosphatidylcholine	Phosphatidylcholines		Level 2
PC aa C36:2	C(18:1;7Z-enoyl/18:1;7Z-enoyl) aa L-Phosphatidylcholine	Phosphatidylcholines		Level 2
PC aa C36:2	C(18:1;8E-enoyl/18:1;8E-enoyl) aa Phosphatidylcholine	Phosphatidylcholines		Level 2
PC aa C36:2	C(18:1;8Z-enoyl/18:1;8Z-enoyl) aa L-Phosphatidylcholine	Phosphatidylcholines		Level 2
PC aa C36:2	C(18:1;9E-enoyl/18:1;9E-enoyl) aa L-Phosphatidylcholine	Phosphatidylcholines		Level 2
PC aa C36:2	C(18:1;9E-enoyl/18:1;9E-enoyl) aa Phosphatidylcholine	Phosphatidylcholines		Level 2
PC aa C36:2	C(18:1;9Z-enoyl/18:1;9Z-enoyl) aa D-Phosphatidylcholine	Phosphatidylcholines		Level 2
PC aa C36:2	PC(18:1(9Z)/18:1(9Z))	Phosphatidylcholines	HMDB00593	Level 2
PC aa C36:2	C(18:2;10Z,12Z-dienoyl/18:0) aa L-Phosphatidylcholine	Phosphatidylcholines		Level 2
PC aa C36:2	C(18:2;2E,4E-dienoyl/18:0) aa L-Phosphatidylcholine	Phosphatidylcholines		Level 2
PC aa C36:2	C(18:2;6Z,9Z-dienoyl/18:0) aa L-Phosphatidylcholine	Phosphatidylcholines		Level 2
PC aa C36:2	C(18:2;6Z,9Z-dienoyl/18:0) aa Phosphatidylcholine	Phosphatidylcholines		Level 2
PC aa C36:2	PC(18:0/18:2(9Z,12Z))	Phosphatidylcholines	HMDB08039	Level 2
PC aa C36:2	C(18:2;9Z,12Z-dienoyl/18:0) aa Phosphatidylcholine	Phosphatidylcholines		Level 2
PC aa C36:2	C(20:2;11E,14E-enoyl/16:0) aa Phosphatidylcholine	Phosphatidylcholines		Level 2
PC aa C36:3	PC(18:0/18:3(6Z,9Z,12Z))	Phosphatidylcholines	HMDB08040	Level 2
PC aa C36:3	C(18:3;9Z,12Z,15Z-trienoyl/18:0) aa Phosphatidylcholine	Phosphatidylcholines		Level 2

PC aa C36:3	C(20:3;11E,14E,17E-trienoyl/16:0) aa Phosphatidylcholine	Phosphatidylcholines		Level 2
PC aa C36:3	C(20:3;5E,8E,11E-trienoyl/16:0) aa Phosphatidylcholine	Phosphatidylcholines		Level 2
PC aa C36:3	PC(16:0/20:3(5Z,8Z,11Z))	Phosphatidylcholines	HMDB07980	Level 2
PC aa C36:3	C(20:3;5Z,8Z,11Z-trienoyl/16:0) aa Phosphatidylcholine	Phosphatidylcholines		Level 2
PC aa C36:3	C(20:3;8E,11E,14E-trienoyl/16:0) aa D-Phosphatidylcholine	Phosphatidylcholines		Level 2
PC aa C36:3	C(20:3;8E,11E,14E-trienoyl/16:0) aa L-Phosphatidylcholine	Phosphatidylcholines		Level 2
PC aa C36:3	C(20:3;8E,11E,14E-trienoyl/16:0) aa Phosphatidylcholine	Phosphatidylcholines		Level 2
PC aa C36:3	PC(16:0/20:3(8Z,11Z,14Z))	Phosphatidylcholines	HMDB07981	Level 2
PC aa C36:3	C(20:3;8Z,11Z,14Z-trienoyl/16:0) aa Phosphatidylcholine	Phosphatidylcholines		Level 2
PC aa C36:3	C18:2;6Z,9Z-dienoyl/18:1;9Z-enoyl) aa L-Phosphatidylcholine	Phosphatidylcholines		Level 2
PC aa C36:3	C18:2;6Z,9Z-dienoyl/18:1;9Z-enoyl) aa Phosphatidylcholine	Phosphatidylcholines		Level 2
PC aa C36:3	PC(18:1(9Z)/18:2(9Z,12Z))	Phosphatidylcholines	HMDB08105	Level 2
PC aa C36:3	C18:2;9Z,12Z-dienoyl/18:1;9Z-enoyl) aa Phosphatidylcholine	Phosphatidylcholines		Level 2
PC aa C36:4	C(16:0/20:4;Z,8E,11E,14E,17E-tetraenoyl) aa L-Phosphatidylcholine	Phosphatidylcholines		Level 2
PC aa C36:4	PC(20:4(5Z,8Z,11Z,14Z)/16:0)	Phosphatidylcholines	HMDB08429	Level 2
PC aa C36:4	C(16:0/20:4;Z,8Z,11Z,14Z-tetraenoyl) aa Phosphatidylcholine	Phosphatidylcholines		Level 2
PC aa C36:4	PC(18:3(6Z,9Z,12Z)/18:1(9Z))	Phosphatidylcholines	HMDB08170	Level 2
PC aa C36:4	PC(18:3(9Z,12Z,15Z)/18:1(9Z))	Phosphatidylcholines	HMDB08203	Level 2
PC aa C36:4	C(18:2;15E,17-dienoyl/18:2;15E,17-dienoyl) aa Phosphatidylcholine	Phosphatidylcholines		Level 2
PC aa C36:4	C(18:2;2E,4E-dienoyl/18:2;2E,17-dienoyl) aa D-Phosphatidylcholine	Phosphatidylcholines		Level 2
PC aa C36:4	C(18:2;2E,4E-dienoyl/18:2;2E,4E-dienoyl) aa D-Phosphatidylcholine	Phosphatidylcholines		Level 2
PC aa C36:4	C(18:2;2E,4E-dienoyl/18:2;2E,4E-dienoyl) aa L-Phosphatidylcholine	Phosphatidylcholines		Level 2
PC aa C36:4	C(18:2;2E,4E-dienoyl/18:2;2E,4E-dienoyl) aa Phosphatidylcholine	Phosphatidylcholines		Level 2
PC aa C36:4	C(18:2;2Z,4Z-dienoyl/18:2;2Z,4Z-dienoyl) aa L-Phosphatidylcholine	Phosphatidylcholines		Level 2
PC aa C36:4	C(18:2;5Z,8Z-dienoyl/18:2;5Z,9Z-dienoyl) aa Phosphatidylcholine	Phosphatidylcholines		Level 2
PC aa C36:4	C(18:2;6Z,9Z-dienoyl/18:2;6Z,9Z-dienoyl) aa L-Phosphatidylcholine	Phosphatidylcholines		Level 2
PC aa C36:4	C(18:2;6Z,9Z-dienoyl/18:2;6Z,9Z-dienoyl) aa Phosphatidylcholine	Phosphatidylcholines		Level 2
PC aa C36:4	C(18:2;9E,12Z-dienoyl/18:2;9E,12Z-dienoyl) aa Phosphatidylcholine	Phosphatidylcholines		Level 2
PC aa C36:4	C(18:2;9Z,11Z-dienoyl/18:2;9Z,11Z-dienoyl) aa L-Phosphatidylcholine	Phosphatidylcholines		Level 2
PC aa C36:4	C(18:2;9Z,12Z-dienoyl/18:2;9Z,12Z-dienoyl) aa D-Phosphatidylcholine	Phosphatidylcholines		Level 2
PC aa C36:4	PC(18:2(9Z,12Z)/18:2(9Z,12Z))	Phosphatidylcholines	HMDB08138	Level 2
PC aa C36:4	C(18:2;9Z,12Z-dienoyl/18:2;9Z,12Z-dienoyl) aa Phosphatidylcholine	Phosphatidylcholines		Level 2
PC aa C36:4	PC(18:1(9Z)/18:3(6Z,9Z,12Z))	Phosphatidylcholines	HMDB08106	Level 2

PC aa C36:4	PC(18:1(9Z)/18:3(9Z,12Z,15Z))	Phosphatidylcholines	HMDB08107	Level 2
PC aa C36:4	C(18:3;9Z,12Z,15Z-trienoyl/18:1;9Z-enoyl) aa Phosphatidylcholine	Phosphatidylcholines		Level 2
PC aa C36:4	C(18:4;5Z,8Z,11Z,14Z-tetraenoyl/18:0) aa Phosphatidylcholine	Phosphatidylcholines		Level 2
PC aa C36:4	PC(18:0/18:4(6Z,9Z,12Z,15Z))	Phosphatidylcholines	HMDB08042	Level 2
PC aa C36:4	C(18:4;9E,11E,13E,15E-tetraenoyl/18:0) aa L-Phosphatidylcholine	Phosphatidylcholines		Level 2
PC aa C36:4	C(18:4;9E,11E,13E,15E-tetraenoyl/18:0) aa Phosphatidylcholine	Phosphatidylcholines		Level 2
PC aa C36:4	C(18:4;9E,11E,13Z,15Z-tetraenoyl/18:0) aa Phosphatidylcholine	Phosphatidylcholines		Level 2
PC aa C36:4	PC(16:0/20:4(5Z,8Z,11Z,14Z))	Phosphatidylcholines	HMDB07982	Level 2
PC aa C36:4	C(20:4;5E,8E,11E,14E-tetraenoyl/16:0) aa Phosphatidylcholine	Phosphatidylcholines		Level 2
PC aa C36:4	C(20:4;5Z,8Z,11Z,14Z-tetraenoyl/16:0) aa L-Phosphatidylcholine	Phosphatidylcholines		Level 2
PC aa C36:4	C(20:4;5Z,8Z,11Z,14Z-tetraenoyl/16:0) aa Phosphatidylcholine	Phosphatidylcholines		Level 2
PC aa C36:5	C(18:1;11Z-enoyl/18:4;2E,4E,6E,11Z-tetraenoyl) aa Phosphatidylcholine	Phosphatidylcholines		Level 2
PC aa C36:5	C(20:4;5Z,8Z,11Z,14Z-tetraenoyl/16:1;7Z-enoyl) aa L-Phosphatidylcholine	Phosphatidylcholines		Level 2
PC aa C36:5	PC(16:1(9Z)/20:4(5Z,8Z,11Z,14Z))	Phosphatidylcholines	HMDB08015	Level 2
PC aa C36:5	C(20:4;5Z,8Z,11Z,14Z-tetraenoyl/16:1;9Z-enoyl) aa Phosphatidylcholine	Phosphatidylcholines		Level 2
PC aa C36:5	C(20:5;5E,8E,11E,14E,17E-pentaenoyl/16:0) aa Phosphatidylcholine	Phosphatidylcholines		Level 2
PC aa C36:5	PC(16:0/20:5(5Z,8Z,11Z,14Z,17Z))	Phosphatidylcholines	HMDB07984	Level 2
PC aa C36:5	C(20:5;5Z,8Z,11Z,14Z,17Z-pentaenoyl/16:0) aa Phosphatidylcholine	Phosphatidylcholines		Level 2
PC aa C36:6	C(18:2;9Z,12Z-dienoyl/18:4;9E,11E,13E,15E-tetraenoyl) aa L-Phosphatidylcholine	Phosphatidylcholines		Level 2
PC aa C36:6	C(18:3;6Z,9Z,12Z-trienoyl/18:3;6Z,9Z,12Z-trienoyl) aa Phosphatidylcholine	Phosphatidylcholines		Level 2
PC aa C36:6	C(18:3;8E,10E,12E-trienoyl/18:3;8E,10E,12E-trienoyl) aa Phosphatidylcholine	Phosphatidylcholines		Level 2
PC aa C36:6	C(18:3;9E,11E,13E-trienoyl/18:3;9E,11E,13E-trienoyl) aa L-Phosphatidylcholine	Phosphatidylcholines		Level 2
PC aa C36:6	C(18:3;9E,11E,13E-trienoyl/18:3;9E,11E,13E-trienoyl) aa Phosphatidylcholine	Phosphatidylcholines		Level 2
PC aa C36:6	C(18:3;9Z,11E,13E-trienoyl/18:3;9Z,11E,13E-trienoyl) aa L-Phosphatidylcholine	Phosphatidylcholines		Level 2
PC aa C36:6	PC(18:3(9Z,12Z,15Z)/18:3(9Z,12Z,15Z))	Phosphatidylcholines	HMDB08206	Level 2
PC aa C36:6	C(18:3;9Z,12Z,15Z-trienoyl/18:3;9Z,12Z,15Z-trienoyl) aa Phosphatidylcholine	Phosphatidylcholines		Level 2
PC aa C36:6	PC(14:0/22:6(4Z,7Z,10Z,13Z,16Z,19Z))	Phosphatidylcholines	HMDB07892	Level 2
PC aa C36:6	C(22:6;4Z,7Z,10Z,13Z,16Z,19Z-hexaenoyl/14:0) aa Phosphatidylcholine	Phosphatidylcholines		Level 2
PC aa C38:0	C(12:0/26:0) aa Phosphatidylcholine	Phosphatidylcholines		Level 2
PC aa C38:0	C(13:0/25:0) aa Phosphatidylcholine	Phosphatidylcholines		Level 2
PC aa C38:0	PC(24:0/14:0)	Phosphatidylcholines	HMDB08755	Level 2
PC aa C38:0	C(15:0/23:0) aa Phosphatidylcholine	Phosphatidylcholines		Level 2
PC aa C38:0	C(16:0/22:0) aa L-Phosphatidylcholine	Phosphatidylcholines		Level 2
PC aa C38:0	PC(22:0/16:0)	Phosphatidylcholines	HMDB08528	Level 2
PC aa C38:0	PC(20:0/18:0)	Phosphatidylcholines	HMDB08267	Level 2

PC aa C38:0	C(19:0/19:0) aa L-Phosphatidylcholine	Phosphatidylcholines		Level 2
PC aa C38:0	C(19:0/19:0) aa Phosphatidylcholine	Phosphatidylcholines		Level 2
PC aa C38:0	PC(18:0/20:0)	Phosphatidylcholines	HMDB08043	Level 2
PC aa C38:0	C(20:0/18:0) aa Phosphatidylcholine	Phosphatidylcholines		Level 2
PC aa C38:0	C(21:0/17:0) aa Phosphatidylcholine	Phosphatidylcholines		Level 2
PC aa C38:0	C(17:0/21:0) aa Phosphatidylcholine	Phosphatidylcholines		Level 2
PC aa C38:0	PC(16:0/22:0)	Phosphatidylcholines	HMDB07985	Level 2
PC aa C38:0	C(22:0/16:0) aa Phosphatidylcholine	Phosphatidylcholines		Level 2
PC aa C38:0	C(23:0/15:0) aa Phosphatidylcholine	Phosphatidylcholines		Level 2
PC aa C38:0	PC(14:0/24:0)	Phosphatidylcholines	HMDB07893	Level 2
PC aa C38:0	C(25:0/13:0) aa L-Phosphatidylcholine	Phosphatidylcholines		Level 2
PC aa C38:1	C(18:1;11E-enoyl/20:0) aa Phosphatidylcholine	Phosphatidylcholines		Level 2
PC aa C38:1	PC(20:0/18:1(11Z))	Phosphatidylcholines	HMDB08268	Level 2
PC aa C38:1	C(18:1;13E-enoyl/20:0) aa Phosphatidylcholine	Phosphatidylcholines		Level 2
PC aa C38:1	C(18:1;6Z-enoyl/20:0) aa L-Phosphatidylcholine	Phosphatidylcholines		Level 2
PC aa C38:1	C(18:1;7Z-enoyl/20:0) aa L-Phosphatidylcholine	Phosphatidylcholines		Level 2
PC aa C38:1	PC(20:0/18:1(9Z))	Phosphatidylcholines	HMDB08269	Level 2
PC aa C38:1	C(18:1;9Z-enoyl/20:0) aa Phosphatidylcholine	Phosphatidylcholines		Level 2
PC aa C38:1	C(20:0/18:1;9E-enoyl) aa L-Phosphatidylcholine	Phosphatidylcholines		Level 2
PC aa C38:1	C(20:0/18:1;9E-enoyl) aa Phosphatidylcholine	Phosphatidylcholines		Level 2
PC aa C38:1	PC(18:1(9Z)/20:0)	Phosphatidylcholines	HMDB08109	Level 2
PC aa C38:1	C(20:0/18:1;9Z-enoyl) aa Phosphatidylcholine	Phosphatidylcholines		Level 2
PC aa C38:1	PC(18:0/20:1(11Z))	Phosphatidylcholines	HMDB08044	Level 2
PC aa C38:1	C(20:1;13Z-enoyl/18:0) aa L-Phosphatidylcholine	Phosphatidylcholines		Level 2
PC aa C38:1	C(20:1;14Z-enoyl/18:0) aa L-Phosphatidylcholine	Phosphatidylcholines		Level 2
PC aa C38:1	C(20:1;5Z-enoyl/18:0) aa L-Phosphatidylcholine	Phosphatidylcholines		Level 2
PC aa C38:1	C(20:1;8Z-enoyl/18:0) aa L-Phosphatidylcholine	Phosphatidylcholines		Level 2
PC aa C38:1	PC(16:0/22:1(13Z))	Phosphatidylcholines	HMDB07986	Level 2
PC aa C38:1	C(22:1;13Z-enoyl/16:0) aa Phosphatidylcholine	Phosphatidylcholines		Level 2
PC aa C38:1	PC(14:0/24:1(15Z))	Phosphatidylcholines	HMDB07894	Level 2
PC aa C38:3	C(20:1;13Z-enoyl/18:2;9Z,12Z-dienoyl) aa L-Phosphatidylcholine	Phosphatidylcholines		Level 2
PC aa C38:3	C(20:3;5E,8E,11E-trienoyl/18:0) aa Phosphatidylcholine	Phosphatidylcholines		Level 2
PC aa C38:3	C(20:3;5Z,11Z,14Z-trienoyl/18:0) aa L-Phosphatidylcholine	Phosphatidylcholines		Level 2
PC aa C38:3	PC(18:0/20:3(5Z,8Z,11Z))	Phosphatidylcholines	HMDB08046	Level 2
PC aa C38:3	C(20:3;5Z,8Z,11Z-trienoyl/18:0) aa Phosphatidylcholine	Phosphatidylcholines		Level 2
PC aa C38:3	C(20:3;5Z,8Z,14Z-trienoyl/18:0) aa L-Phosphatidylcholine	Phosphatidylcholines		Level 2
PC aa C38:3	PC(18:0/20:3(8Z,11Z,14Z))	Phosphatidylcholines	HMDB08047	Level 2
PC aa C38:3	C(20:3;8Z,11Z,14Z-trienoyl/18:0) aa Phosphatidylcholine	Phosphatidylcholines		Level 2

PC aa C38:3	C(22:3;13Z,16Z,19Z-trienoyl/16:0) aa Phosphatidylcholine	Phosphatidylcholines		Level 2
PC aa C38:4	PC(18:1(9Z)/20:3(5Z,8Z,11Z))	Phosphatidylcholines	HMDB08112	Level 2
PC aa C38:4	C(20:4;5E,8E,11E,14E-tetraenoyl/18:0) aa Phosphatidylcholine	Phosphatidylcholines		Level 2
PC aa C38:4	PC(18:0/20:4(5Z,8Z,11Z,14Z))	Phosphatidylcholines	HMDB08048	Level 2
PC aa C38:4	C(20:4;5Z,8Z,11Z,14Z-tetraenoyl/18:0) aa Phosphatidylcholine	Phosphatidylcholines		Level 2
PC aa C38:4	C(20:4;8Z,10Z,12Z,14Z-tetraenoyl/18:0) aa L-Phosphatidylcholine	Phosphatidylcholines		Level 2
PC aa C38:4	PC(16:0/22:4(7Z,10Z,13Z,16Z))	Phosphatidylcholines	HMDB07988	Level 2
PC aa C38:4	C(22:4;7Z,10Z,13Z,16Z-tetraenoyl/16:0) aa Phosphatidylcholine	Phosphatidylcholines		Level 2
PC aa C38:5	PC(18:1(9Z)/20:4(5Z,8Z,11Z,14Z))	Phosphatidylcholines	HMDB08114	Level 2
PC aa C38:5	C(20:4;5Z,8Z,11Z,14Z-tetraenoyl/18:1;9Z-enoyl) aa Phosphatidylcholine	Phosphatidylcholines		Level 2
PC aa C38:5	PC(18:0/20:5(5Z,8Z,11Z,14Z,17Z))	Phosphatidylcholines	HMDB08050	Level 2
PC aa C38:5	C(20:5;5Z,8Z,11Z,14Z,17Z-pentaenoyl/18:0) aa Phosphatidylcholine	Phosphatidylcholines		Level 2
PC aa C38:5	C(20:5;9Z,11Z,13Z,15Z,17Z-pentaenoyl/18:0) aa L-Phosphatidylcholine	Phosphatidylcholines		Level 2
PC aa C38:5	C(22:5;4E,7E,10E,13E,16E-pentaenoyl/16:0) aa Phosphatidylcholine	Phosphatidylcholines		Level 2
PC aa C38:5	PC(16:0/22:5(4Z,7Z,10Z,13Z,16Z))	Phosphatidylcholines	HMDB07989	Level 2
PC aa C38:5	C(22:5;4Z,7Z,10Z,13Z,16Z-pentaenoyl/16:0) aa Phosphatidylcholine	Phosphatidylcholines		Level 2
PC aa C38:5	PC(16:0/22:5(7Z,10Z,13Z,16Z,19Z))	Phosphatidylcholines	HMDB07990	Level 2
PC aa C38:5	C(22:5;7Z,10Z,13Z,16Z,19Z-pentaenoyl/16:0) aa Phosphatidylcholine	Phosphatidylcholines		Level 2
PC aa C38:6	C(16:0/22:6;4E,7E,10E,13E,16E,19E-hexaenoyl) aa L-Phosphatidylcholine	Phosphatidylcholines		Level 2
PC aa C38:6	PC(22:6(4Z,7Z,10Z,13Z,16Z,19Z)/16:0)	Phosphatidylcholines	HMDB08725	Level 2
PC aa C38:6	C(16:0/22:6;4Z,7Z,10Z,13Z,16Z,19Z-hexaenoyl) aa Phosphatidylcholine	Phosphatidylcholines		Level 2
PC aa C38:6	PC(20:5(5Z,8Z,11Z,14Z,17Z)/18:1(9Z))	Phosphatidylcholines	HMDB08499	Level 2
PC aa C38:6	PC(20:4(5Z,8Z,11Z,14Z)/18:2(9Z,12Z))	Phosphatidylcholines	HMDB08434	Level 2
PC aa C38:6	PC(18:2(9Z,12Z)/20:4(5Z,8Z,11Z,14Z))	Phosphatidylcholines	HMDB08147	Level 2
PC aa C38:6	C(20:4;5Z,8Z,11Z,14Z-tetraenoyl/18:2;9Z,12Z-dienoyl) aa Phosphatidylcholine	Phosphatidylcholines		Level 2
PC aa C38:6	PC(18:1(11Z)/20:5(5Z,8Z,11Z,14Z,17Z))	Phosphatidylcholines	HMDB08083	Level 2
PC aa C38:6	PC(18:1(9Z)/20:5(5Z,8Z,11Z,14Z,17Z))	Phosphatidylcholines	HMDB08116	Level 2
PC aa C38:6	C(20:5;5Z,8Z,11Z,14Z,17Z-pentaenoyl/18:1;9Z-enoyl) aa Phosphatidylcholine	Phosphatidylcholines		Level 2
PC aa C38:6	C(22:6;3Z,6Z,9Z,12Z,15Z,18E-hexaenoyl/16:0) aa Phosphatidylcholine	Phosphatidylcholines		Level 2
PC aa C38:6	C(22:6;4E,7E,10E,13E,16E,19E-hexaenoyl/16:0) aa L-Phosphatidylcholine	Phosphatidylcholines		Level 2
PC aa C38:6	C(22:6;4E,7E,10E,13E,16E,19E-hexaenoyl/16:0) aa Phosphatidylcholine	Phosphatidylcholines		Level 2
PC aa C38:6	C(22:6;4Z,7Z,10Z,13Z,16Z,19Z-hexaenoyl/16:0) aa D-Phosphatidylcholine	Phosphatidylcholines		Level 2
PC aa C38:6	PC(16:0/22:6(4Z,7Z,10Z,13Z,16Z,19Z))	Phosphatidylcholines	HMDB07991	Level 2
PC aa C38:6	C(22:6;4Z,7Z,10Z,13Z,16Z,19Z-hexaenoyl/16:0) aa Phosphatidylcholine	Phosphatidylcholines		Level 2

PC aa C40:1	PC(20:0/20:1(11Z))	Phosphatidylcholines	HMDB08275	Level 2
PC aa C40:1	C(20:1;13Z-enoyl/20:0) aa L-Phosphatidylcholine	Phosphatidylcholines		Level 2
PC aa C40:1	C(20:1;5Z-enoyl/20:0) aa L-Phosphatidylcholine	Phosphatidylcholines		Level 2
PC aa C40:1	C(20:1;8Z-enoyl/20:0) aa L-Phosphatidylcholine	Phosphatidylcholines		Level 2
PC aa C40:1	C(22:0/18:1;11E-enoyl) aa L-Phosphatidylcholine	Phosphatidylcholines		Level 2
PC aa C40:1	PC(18:1(11Z)/22:0)	Phosphatidylcholines	HMDB08084	Level 2
PC aa C40:1	C(22:0/18:1;9E-enoyl) aa Phosphatidylcholine	Phosphatidylcholines		Level 2
PC aa C40:1	PC(18:1(9Z)/22:0)	Phosphatidylcholines	HMDB08117	Level 2
PC aa C40:1	C(22:0/18:1;9Z-enoyl) aa Phosphatidylcholine	Phosphatidylcholines		Level 2
PC aa C40:1	PC(18:0/22:1(13Z))	Phosphatidylcholines	HMDB08052	Level 2
PC aa C40:1	C(24:1;E15-enoyl/16:0) aa Phosphatidylcholine	Phosphatidylcholines		Level 2
PC aa C40:1	C(24:1;Z15-enoyl/16:0) aa D-Phosphatidylcholine	Phosphatidylcholines		Level 2
PC aa C40:1	PC(16:0/24:1(15Z))	Phosphatidylcholines	HMDB07993	Level 2
PC aa C40:1	C(24:1;Z15-enoyl/16:0) aa Phosphatidylcholine	Phosphatidylcholines		Level 2
PC aa C40:2	C(20:1;11E-enoyl/20:1;11E-enoyl) aa L-Phosphatidylcholine	Phosphatidylcholines		Level 2
PC aa C40:2	PC(20:1(11Z)/20:1(11Z))	Phosphatidylcholines	HMDB08308	Level 2
PC aa C40:2	C(20:1;11Z-enoyl/20:1;11Z-enoyl) aa Phosphatidylcholine	Phosphatidylcholines		Level 2
PC aa C40:2	C(20:1;9E-enoyl/20:1;9E-enoyl) aa L-Phosphatidylcholine	Phosphatidylcholines		Level 2
PC aa C40:2	C(20:1;9Z-enoyl/20:1;9Z-enoyl) aa L-Phosphatidylcholine	Phosphatidylcholines		Level 2
PC aa C40:2	C(20:1;9Z-enoyl/20:1;9Z-enoyl) aa Phosphatidylcholine	Phosphatidylcholines		Level 2
PC aa C40:2	PC(20:0/20:2(11Z,14Z))	Phosphatidylcholines	HMDB08276	Level 2
PC aa C40:3	C(20:3;11Z,14Z.17Z-trienoyl/20:0) aa L-Phosphatidylcholine	Phosphatidylcholines		Level 2
PC aa C40:3	C(22:3;10Z,13Z,16Z-trienoyl/18:0) aa L-Phosphatidylcholine	Phosphatidylcholines		Level 2
PC aa C40:4	PC(22:4(7Z,10Z,13Z,16Z)/18:0)	Phosphatidylcholines	HMDB08628	Level 2
PC aa C40:4	C(20:2;11E,14E-dienoyl/20:2;11E,14E-dienoyl) aa L-Phosphatidylcholine	Phosphatidylcholines		Level 2
PC aa C40:4	C(20:2;2E,4E-dienoyl/20:2;2E,4E-dienoyl) aa D-Phosphatidylcholine	Phosphatidylcholines		Level 2
PC aa C40:4	PC(20:0/20:4(5Z,8Z,11Z,14Z))	Phosphatidylcholines	HMDB08279	Level 2
PC aa C40:4	PC(18:0/22:4(7Z,10Z,13Z,16Z))	Phosphatidylcholines	HMDB08054	Level 2
PC aa C40:4	C(22:3;7Z10Z,13Z,16Z-tetraenoyl/18:0) aa Phosphatidylcholine	Phosphatidylcholines		Level 2
PC aa C40:5	PC(18:1(9Z)/22:4(7Z,10Z,13Z,16Z))	Phosphatidylcholines	HMDB08120	Level 2
PC aa C40:5	C(22:5;4E,7E,10E,13E,16E-pentaenoyl/18:0) aa Phosphatidylcholine	Phosphatidylcholines		Level 2
PC aa C40:5	PC(18:0/22:5(4Z,7Z,10Z,13Z,16Z))	Phosphatidylcholines	HMDB08055	Level 2
PC aa C40:5	C(22:5;4Z,7Z,10Z,13Z,16Z-pentaenoyl/18:0) aa Phosphatidylcholine	Phosphatidylcholines		Level 2
PC aa C40:5	PC(18:0/22:5(7Z,10Z,13Z,16Z,19Z))	Phosphatidylcholines	HMDB08056	Level 2
PC aa C40:5	C(22:5;7Z,10Z,13Z,16Z,19Z-pentaenoyl/18:0) aa Phosphatidylcholine	Phosphatidylcholines		Level 2
PC aa C40:6	PC(18:1(11Z)/22:5(7Z,10Z,13Z,16Z,19Z))	Phosphatidylcholines	HMDB08089	Level 2

PC aa C40:6	PC(18:1(9Z)/22:5(7Z,10Z,13Z,16Z,19Z))	Phosphatidylcholines	HMDB08122	Level 2
PC aa C40:6	C(22:6;4E,7E,10E,13E,16E,19E-hexaenoyl/18:0) aa Phosphatidylcholine	Phosphatidylcholines		Level 2
PC aa C40:6	PC(18:0/22:6(4Z,7Z,10Z,13Z,16Z,19Z))	Phosphatidylcholines	HMDB08057	Level 2
PC aa C40:6	C(22:6;4Z,7Z,10Z,13Z,16Z,19Z-hexaenoyl/18:0) aa Phosphatidylcholine	Phosphatidylcholines		Level 2
PC aa C40:6	C(22:6;9Z,11Z,13Z,15Z,17Z,19E-hexaenoyl/18:0) aa L-Phosphatidylcholine	Phosphatidylcholines		Level 2
PC aa C42:0	C(16:0/26:0) aa L-Phosphatidylcholine	Phosphatidylcholines		Level 2
PC aa C42:0	C(17:0/25:0) aa L-Phosphatidylcholine	Phosphatidylcholines		Level 2
PC aa C42:0	PC(24:0/18:0)	Phosphatidylcholines	HMDB08760	Level 2
PC aa C42:0	C(19:0/23:0) aa Phosphatidylcholine	Phosphatidylcholines		Level 2
PC aa C42:0	PC(22:0/20:0)	Phosphatidylcholines	HMDB08537	Level 2
PC aa C42:0	C(21:0/21:0) aa L-Phosphatidylcholine	Phosphatidylcholines		Level 2
PC aa C42:0	C(21:0/21:0) aa Phosphatidylcholine	Phosphatidylcholines		Level 2
PC aa C42:0	PC(20:0/22:0)	Phosphatidylcholines	HMDB08282	Level 2
PC aa C42:0	C(23:0/19:0) aa Phosphatidylcholine	Phosphatidylcholines		Level 2
PC aa C42:0	PC(18:0/24:0)	Phosphatidylcholines	HMDB08058	Level 2
PC aa C42:0	C(25:0/17:0) aa Phosphatidylcholine	Phosphatidylcholines		Level 2
PC aa C42:0	C(26:0/16:0) aa L-Phosphatidylcholine	Phosphatidylcholines		Level 2
PC aa C42:0	C(26:0/16:0) aa Phosphatidylcholine	Phosphatidylcholines		Level 2
PC aa C42:1	C(18:1;6Z-enoyl/24:0) aa L-Phosphatidylcholine	Phosphatidylcholines		Level 2
PC aa C42:1	PC(24:0/18:1(9Z))	Phosphatidylcholines	HMDB08762	Level 2
PC aa C42:1	PC(22:0/20:1(11Z))	Phosphatidylcholines	HMDB08538	Level 2
PC aa C42:1	C(22:0/20:1;11E-enoyl) aa L-Phosphatidylcholine	Phosphatidylcholines		Level 2
PC aa C42:1	C(22:0/20:1;11Z-enoyl) aa L-Phosphatidylcholine	Phosphatidylcholines		Level 2
PC aa C42:1	PC(20:0/22:1(13Z))	Phosphatidylcholines	HMDB08283	Level 2
PC aa C42:1	PC(18:1(9Z)/24:0)	Phosphatidylcholines	HMDB08124	Level 2
PC aa C42:1	PC(18:0/24:1(15Z))	Phosphatidylcholines	HMDB08059	Level 2
PC aa C42:2	C(21:1;12Z-enoyl/21:1;12Z-enoyl) aa Phosphatidylcholine	Phosphatidylcholines		Level 2
PC aa C42:2	C(26:2;5Z,9Z-dienoyl/16:0) aa L-Phosphatidylcholine	Phosphatidylcholines		Level 2
PC aa C42:2	C16:0/26:1;5Z,9Z-dienoyl) aa L-Phosphatidylcholine	Phosphatidylcholines		Level 2
PC aa C42:4	Phosphatidylcholine with diacyl residue sum C42:4	Phosphatidylcholines		Level 2
PC aa C42:5	PC(20:0/22:5(7Z,10Z,13Z,16Z,19Z))	Phosphatidylcholines	HMDB08287	Level 2
PC aa C42:6	C(22:6;4Z,7Z,10Z,13Z,16Z,19Z-hexaenoyl/20:0) aa Phosphatidylcholine	Phosphatidylcholines		Level 2
PC aa C42:6	PC(20:0/22:6(4Z,7Z,10Z,13Z,16Z,19Z))	Phosphatidylcholines	HMDB08288	Level 2
PC ae C30:0	C(16:0/e14:0) ae Phosphatidylcholine	Phosphatidylcholines		Level 2
PC ae C30:0	PC(o-14:0/16:0)	Phosphatidylcholines	HMDB13341	Level 2
PC ae C30:1	PC(o-14:0/16:1(9Z))	Phosphatidylcholines	HMDB13402	Level 2
PC ae C30:2	Phosphatidylcholine with acyl-alkyl residue sum C30:2	Phosphatidylcholines		Level 2
PC ae C32:1	C(18:1;9Z-enoyl/e14:0) ae L-Phosphatidylcholine	Phosphatidylcholines		Level 2

PC ae C32:2	C(18:1;9Z-enoyl/e14:1;1Z-en) ae L-Phosphatidylcholine	Phosphatidylcholines		Level 2
PC ae C32:2	C(18:2;9Z,12Z-dienoyl/e14:0) ae L-Phosphatidylcholine	Phosphatidylcholines		Level 2
PC ae C34:0	C(16:0/e18:0) ae D-Phosphatidylcholine	Phosphatidylcholines		Level 2
PC ae C34:0	C(16:0/e18:0) ae L-Phosphatidylcholine	Phosphatidylcholines		Level 2
PC ae C34:0	C(16:0/e18:0) ae Phosphatidylcholine	Phosphatidylcholines		Level 2
PC ae C34:0	C(17:0/e17:0) ae L-Phosphatidylcholine	Phosphatidylcholines		Level 2
PC ae C34:0	C(18:0/e16:0) ae D-Phosphatidylcholine	Phosphatidylcholines		Level 2
PC ae C34:0	PC(o-16:0/18:0)	Phosphatidylcholines	HMDB13405	Level 2
PC ae C34:0	C(18:0/e16:0) ae Phosphatidylcholine	Phosphatidylcholines		Level 2
PC ae C34:0	C(e16:0/18:0) ae Phosphatidylcholine	Phosphatidylcholines		Level 2
PC ae C34:1	C(16:0;e18:1;9Z-en) ae Phosphatidylcholine	Phosphatidylcholines		Level 2
PC ae C34:1	C(16:1;9Z-enoyl/e18:0) ae L-Phosphatidylcholine	Phosphatidylcholines		Level 2
PC ae C34:1	C(18:1;9E-enoyl/e16:0) ae Phosphatidylcholine	Phosphatidylcholines		Level 2
PC ae C34:1	C(18:1;9Z-enoyl/e16:0) ae D-Phosphatidylcholine	Phosphatidylcholines		Level 2
PC ae C34:1	C(18:1;9Z-enoyl/e16:0) ae L-Phosphatidylcholine	Phosphatidylcholines		Level 2
PC ae C34:1	C(18:1;9Z-enoyl/e16:0) ae Phosphatidylcholine	Phosphatidylcholines		Level 2
PC ae C34:2	PC(O-16:0/18:2(9Z,12Z))	Phosphatidylcholines	HMDB11151	Level 2
PC ae C34:2	C(18:1;9Z,12Z-dienoyl/e16:0) ae Phosphatidylcholine	Phosphatidylcholines		Level 2
PC ae C34:2	C(18:1;9Z-enoyl/e16:1;1E-en) ae Phosphatidylcholine	Phosphatidylcholines		Level 2
PC ae C34:2	C(18:1;9Z-enoyl/e16:1;1Z-en) ae L-Phosphatidylcholine	Phosphatidylcholines		Level 2
PC ae C34:2	C(18:1;9Z-enoyl/e16:1;1Z-en) ae Phosphatidylcholine	Phosphatidylcholines		Level 2
PC ae C34:3	C(18:1;6Z,9Z,12Z-trienoyl/e16:0) ae L-Phosphatidylcholine	Phosphatidylcholines		Level 2
PC ae C34:3	C(18:1;9Z,12Z,15Z-trienoyl/e16:0) ae D-Phosphatidylcholine	Phosphatidylcholines		Level 2
PC ae C34:3	C(18:1;9Z,12Z,15Z-trienoyl/e16:0) ae L-Phosphatidylcholine	Phosphatidylcholines		Level 2
PC ae C34:3	PC(P-16:0/18:2(9Z,12Z))	Phosphatidylcholines	HMDB11211	Level 2
PC ae C36:0	C(16:0/e20:0) ae L-Phosphatidylcholine	Phosphatidylcholines		Level 2
PC ae C36:0	C(18:0/e18:0) ae D-Phosphatidylcholine	Phosphatidylcholines		Level 2
PC ae C36:0	PC(o-18:0/18:0)	Phosphatidylcholines	HMDB13417	Level 2
PC ae C36:0	C(18:0/e18:0) ae Phosphatidylcholine	Phosphatidylcholines		Level 2
PC ae C36:0	PC(o-16:0/20:0)	Phosphatidylcholines	HMDB13406	Level 2
PC ae C36:0	C(22:0/e14:0) ae L-Phosphatidylcholine	Phosphatidylcholines		Level 2
PC ae C36:1	C(20:1;9Z-enoyl/e16:0) ae L-Phosphatidylcholine	Phosphatidylcholines		Level 2
PC ae C36:2	PC(P-18:0/18:1(9Z))	Phosphatidylcholines	HMDB11243	Level 2
PC ae C36:3	C(20:3;8Z,11Z,14Z-trienoyl/e16:0) ae L-Phosphatidylcholine	Phosphatidylcholines		Level 2
PC ae C36:4	C(20:4;5E,8E,11E,14E-tetraenoyl/e16:0) ae L-Phosphatidylcholine	Phosphatidylcholines		Level 2
PC ae C36:4	C(20:4;5E,8E,11E,14E-tetraenoyl/e16:0) ae Phosphatidylcholine	Phosphatidylcholines		Level 2
PC ae C36:4	C(20:4;5Z,8Z,11Z,14Z-tetraenoyl/e16:0) ae L-	Phosphatidylcholines		Level 2

	Phosphatidylcholine			
PC ae C36:4	C(20:4;5Z,8Z,11Z,14Z-tetraenoyl/e16:0) ae Phosphatidylcholine	Phosphatidylcholines		Level 2
PC ae C36:5	PC(P-16:0/20:4(5Z,8Z,11Z,14Z))	Phosphatidylcholines	HMDB11220	Level 2
PC ae C36:5	C(20:5;(5Z,8Z,11Z,14Z,17Z-pentaenoyl/e16:0) ae L-Phosphatidylcholine	Phosphatidylcholines		Level 2
PC ae C38:0	PC(o-18:0/20:0)	Phosphatidylcholines	HMDB13419	Level 2
PC ae C38:0	C(20:0/e18:0) ae Phosphatidylcholine	Phosphatidylcholines		Level 2
PC ae C38:0	PC(o-16:0/22:0)	Phosphatidylcholines	HMDB13408	Level 2
PC ae C38:0	C(22:0/e16:0) ae Phosphatidylcholine	Phosphatidylcholines		Level 2
PC ae C38:1	C(20:1;9Z-enoyl/e18:0) ae L-Phosphatidylcholine	Phosphatidylcholines		Level 2
PC ae C38:2	Phosphatidylcholine with acyl-alkyl residue sum C38:2	Phosphatidylcholines		Level 2
PC ae C38:3	Phosphatidylcholine with acyl-alkyl residue sum C38:3	Phosphatidylcholines		Level 2
PC ae C38:4	C(20:4;5E,8E,11E,14E-tetraenoyl/e18:0) ae L-Phosphatidylcholine	Phosphatidylcholines		Level 2
PC ae C38:4	C(20:4;5E,8E,11E,14E-tetraenoyl/e18:0) ae Phosphatidylcholine	Phosphatidylcholines		Level 2
PC ae C38:4	PC(o-18:0/20:4(8Z,11Z,14Z,17Z))	Phosphatidylcholines	HMDB13420	Level 2
PC ae C38:4	C(20:4;5Z,8Z,11Z,14Z-tetraenoyl/e18:0) ae Phosphatidylcholine	Phosphatidylcholines		Level 2
PC ae C38:4	C(22:4;7Z,10Z,13Z,16Z-tetraenoyl/e16:0) ae Phosphatidylcholine	Phosphatidylcholines		Level 2
PC ae C38:5	PC(o-18:1(9Z)/20:4(8Z,11Z,14Z,17Z))	Phosphatidylcholines	HMDB13432	Level 2
PC ae C38:5	C(20:4;5Z,8Z,11Z,14Z-tetraenoyl/e18:1;1Z-en) ae L-Phosphatidylcholine	Phosphatidylcholines		Level 2
PC ae C38:5	C(20:5;5Z,8Z,11Z,14Z,17Z-pentaenoyl/e18:0) ae L-Phosphatidylcholine	Phosphatidylcholines		Level 2
PC ae C38:5	C(22:5;7Z,10Z,13Z,16Z,19Z-pentaenoyl/e16:0) ae L-Phosphatidylcholine	Phosphatidylcholines		Level 2
PC ae C38:5	C(22:5;7Z,10Z,13Z,16Z,19Z-pentaenoyl/e16:0) ae Phosphatidylcholine	Phosphatidylcholines		Level 2
PC ae C38:6	PC(o-16:0/22:6(4Z,7Z,10Z,13Z,16Z,19Z))	Phosphatidylcholines	HMDB13409	Level 2
PC ae C38:6	C(22:6;4Z,7Z,10Z,13Z,16Z,19Z-pentaenoyl/e16:0) ae Phosphatidylcholine	Phosphatidylcholines		Level 2
PC ae C40:0	PC(o-18:0/22:0)	Phosphatidylcholines	HMDB13421	Level 2
PC ae C40:0	C(22:0/e18:0) ae Phosphatidylcholine	Phosphatidylcholines		Level 2
PC ae C40:1	Phosphatidylcholine with acyl-alkyl residue sum C40:1	Phosphatidylcholines		Level 2
PC ae C40:2	Phosphatidylcholine with acyl-alkyl residue sum C40:2	Phosphatidylcholines		Level 2
PC ae C40:3	Phosphatidylcholine with acyl-alkyl residue sum C40:3	Phosphatidylcholines		Level 2
PC ae C40:4	Phosphatidylcholine with acyl-alkyl residue sum C40:4	Phosphatidylcholines		Level 2
PC ae C40:5	C(22:5;4Z,7Z,10Z,13Z,16Z-pentaenoyl/e18:0) ae L-Phosphatidylcholine	Phosphatidylcholines		Level 2
PC ae C40:5	C(22:5;7E,10E,13E,16E,19E-pentaenoyl/e18:0) ae Phosphatidylcholine	Phosphatidylcholines		Level 2
PC ae C40:5	C(22:5;7Z,10Z,13Z,16Z,19Z-pentaenoyl/e18:0) ae L-Phosphatidylcholine	Phosphatidylcholines		Level 2
PC ae C40:6	PC(o-18:0/22:6(4Z,7Z,10Z,13Z,16Z,19Z))	Phosphatidylcholines	HMDB13422	Level 2
PC ae C40:6	C(22:5;4Z,7Z,10Z,13Z,16Z,19Z-hexaenoyl/e18:0) ae Phosphatidylcholine	Phosphatidylcholines		Level 2

PC ae C42:0	PC(o-20:0/22:0)	Phosphatidylcholines	HMDB13443	Level 2
PC ae C42:1	Phosphatidylcholine with acyl-alkyl residue sum C42:1	Phosphatidylcholines		Level 2
PC ae C42:2	Phosphatidylcholine with acyl-alkyl residue sum C42:2	Phosphatidylcholines		Level 2
PC ae C42:3	Phosphatidylcholine with acyl-alkyl residue sum C42:3	Phosphatidylcholines		Level 2
PC ae C42:4	Phosphatidylcholine with acyl-alkyl residue sum C42:4	Phosphatidylcholines		Level 2
PC ae C42:5	Phosphatidylcholine with acyl-alkyl residue sum C42:5	Phosphatidylcholines		Level 2
PC ae C44:3	Phosphatidylcholine with acyl-alkyl residue sum C44:3	Phosphatidylcholines		Level 2
PC ae C44:4	Phosphatidylcholine with acyl-alkyl residue sum C44:4	Phosphatidylcholines		Level 2
PC ae C44:5	Phosphatidylcholine with acyl-alkyl residue sum C44:5	Phosphatidylcholines		Level 2
PC ae C44:6	Phosphatidylcholine with acyl-alkyl residue sum C44:6	Phosphatidylcholines		Level 2
SM (OH) C14:1	Hydroxysphingomyelin with acyl residue sum C14:1	Sphingomyelins		Level 2
SM (OH) C16:1	Hydroxysphingomyelin with acyl residue sum C16:1	Sphingomyelins		Level 2
SM (OH) C22:1	Hydroxysphingomyelin with acyl residue sum C22:1	Sphingomyelins		Level 2
SM (OH) C22:2	Hydroxysphingomyelin with acyl residue sum C22:2	Sphingomyelins		Level 2
SM (OH) C24:1	Hydroxysphingomyelin with acyl residue sum C24:1	Sphingomyelins		Level 2
SM C16:0	C16:0 Sphingomyelin	Sphingomyelins		Level 2
SM C16:1	Sphingomyelin with acyl residue sum C16:1	Sphingomyelins		Level 2
SM C18:0	C18:0 Sphingomyelin	Sphingomyelins		Level 2
SM C18:1	C18:1;11Z-enoyl Sphingomyelin	Sphingomyelins	HMDB12100	Level 2
SM C18:1	C18:1;9Z-enoyl Sphingomyelin	Sphingomyelins	HMDB12101	Level 2
SM C20:2	Sphingomyelin with acyl residue sum C20:2	Sphingomyelins		Level 2
SM C22:3	Sphingomyelin with acyl residue sum C22:3	Sphingomyelins		Level 2
SM C24:0	C24:0 Sphingomyelin	Sphingomyelins		Level 2
SM C24:1	SM(d18:1/24:1(15Z))	Sphingomyelins	HMDB12107	Level 2
SM C26:0	C26:0 Sphingomyelin	Sphingomyelins		Level 2
SM C26:1	SM(d18:0/26:1(17Z))	Sphingomyelins	HMDB13461	Level 2
H1	Aldohexose	Hexoses		Level 2
H1	D-Galactose	Hexoses	HMDB00143	Level 2
H1	Alpha-D-Glucose	Hexoses	HMDB03345	Level 2
H1	alpha-D-Mannopyranose	Hexoses		Level 2
H1	Alpha-L-Galactopyranose	Hexoses		Level 2
H1	Beta-D-Galactose	Hexoses	HMDB03449	Level 2
H1	Beta-D-Glucose	Hexoses	HMDB00516	Level 2
H1	D-Allopyranose	Hexoses		Level 2
H1	D-Allose	Hexoses		Level 2
H1	D-Altropyranose	Hexoses		Level 2
H1	D-Altrose	Hexoses		Level 2

H1	D-Erythrohexulose	Hexoses		Level 2
H1	D-Fructose	Hexoses	HMDB00660	Level 2
H1	D-Fructopyranose	Hexoses		Level 2
H1	D-Fructose	Hexoses		Level 2
H1	D-Galactose	Hexoses		Level 2
H1	D-Glucose	Hexoses	HMDB00122	Level 2
H1	D-Gulopyranose	Hexoses		Level 2
H1	D-Gulose	Hexoses		Level 2
H1	D-Idopyranose	Hexoses		Level 2
H1	D-Idose	Hexoses		Level 2
H1	D-Mannose	Hexoses	HMDB00169	Level 2
H1	D-Psicopyranose	Hexoses		Level 2
H1	D-Psicose	Hexoses		Level 2
H1	D-Sorbopyranose	Hexoses		Level 2
H1	D-Sorbose	Hexoses		Level 2
H1	D-Tagatofuranose	Hexoses		Level 2
H1	D-Tagatopyranose	Hexoses		Level 2
H1	D-Tagatose	Hexoses	HMDB03418	Level 2
H1	D-Talopyranose	Hexoses		Level 2
H1	D-Talose	Hexoses		Level 2
H1	Gulose	Hexoses		Level 2
H1	Ketohexose	Hexoses		Level 2
H1	L-Allopyranose	Hexoses		Level 2
H1	L-Allose	Hexoses		Level 2
H1	L-Altrose	Hexoses		Level 2
H1	L-Fructofuranose	Hexoses		Level 2
H1	L-Fructopyranose	Hexoses		Level 2
H1	L-Galactose	Hexoses		Level 2
H1	L-Glucose	Hexoses		Level 2
H1	L-Gulose	Hexoses	HMDB12326	Level 2
H1	L-Gulose	Hexoses		Level 2
H1	L-Idose	Hexoses		Level 2
H1	L-Mannopyranose	Hexoses		Level 2
H1	L-Mannose	Hexoses		Level 2
H1	L-Sorbopyranose	Hexoses		Level 2
H1	L-Sorbose	Hexoses		Level 2
H1	L-Talose	Hexoses		Level 2

Table S-2 List of the slopes of the linear regression between cell number and analyte concentration. We harvested different cell amounts containing cell numbers within the linear range of the DNA quantification method by trypsinization and performed targeted metabolomics as described in the materials and methods section. Correlation between the cell number and the metabolite concentration was analyzed. The table lists the slopes of the linear regression analysis for the metabolites fulfilling the LOD selection criterion.

Metabolite	Hep G2	THLE-2	HK-2	SGBS
C0	NA	NA	NA	NA
C10	NA	NA	NA	NA
C10:1	NA	NA	NA	NA
C10:2	NA	NA	NA	NA
C12	NA	NA	NA	NA
C12-DC	NA	NA	NA	NA
C12:1	NA	NA	NA	NA
C14	NA	NA	NA	NA
C14:1	NA	NA	NA	NA
C14:1-OH	NA	NA	NA	NA
C14:2	NA	NA	NA	NA
C14:2-OH	NA	NA	NA	NA
C16	NA	4.1225E-07	NA	9.1786E-08
C16-OH	NA	NA	NA	NA
C16:1	-6.77E-09	1.2215E-08	-3.38E-08	-1.4872E-08
C16:1-OH	NA	NA	NA	NA
C16:2	-6.53E-10	NA	NA	NA
C16:2-OH	NA	NA	NA	NA
C18	NA	NA	NA	NA
C18:1	NA	1.6168E-07	NA	NA
C18:1-OH	NA	NA	NA	NA
C18:2	NA	NA	NA	NA
C2	NA	2.9657E-07	NA	NA
C3	NA	3.3E-07	NA	NA
C3-DC (C4-OH)	NA	NA	NA	NA
C3-OH	NA	NA	NA	NA
C3:1	NA	NA	NA	NA
C4	NA	1.9596E-07	NA	7.3357E-08
C4 1	NA	NA	NA	NA
C5	NA	7.9955E-08	NA	NA
C5-DC (C6-OH)	NA	NA	NA	NA
C5-M-DC	NA	NA	NA	NA
C5-OH (C3-DC-M)	NA	NA	NA	NA
C5:1	NA	NA	NA	NA
C5:1-DC	NA	2.3191E-09	NA	NA

C6 (C4:1-DC)	NA	NA	NA	NA
C6:1	NA	NA	NA	NA
C7-DC	NA	NA	NA	NA
C8	NA	NA	NA	NA
C9	NA	NA	NA	NA
Ala	1.76E-05	3.0355E-05	3.36E-05	8.3465E-05
Arg	NA	2.7686E-05	2.34E-05	7.9498E-06
Asn	NA	NA	NA	9.5302E-06
Asp	NA	9.5588E-05	2.16E-05	3.9667E-05
Cit	NA	NA	NA	NA
Gln	NA	2.3686E-05	NA	NA
Glu	6.62E-05	0.00024702	0.00025459	0.0007705
Gly	NA	6.919E-05	6.94E-05	0.00016107
His	NA	4.8306E-06	2.61E-06	5.049E-06
Ile	NA	4.8334E-06	1.31E-06	7.3481E-06
Leu	2.62E-06	1.0704E-05	6.38E-06	1.0388E-05
Lys	NA	1.5547E-05	6.35E-06	8.9042E-06
Met	7.33E-07	3.5482E-06	NA	3.8255E-06
Orn	NA	NA	6.09E-06	NA
Phe	NA	6.3481E-06	NA	8.6552E-06
Pro	2.19E-05	4.4434E-05	9.79E-05	0.00012083
Ser	4.53E-06	3.405E-05	1.46E-05	3.0479E-05
Thr	4.44E-06	1.309E-05	3.17E-06	1.7309E-05
Trp	NA	NA	NA	NA
Tyr	NA	NA	NA	9.5694E-06
Val	3.11E-06	1.1163E-05	NA	1.0556E-05
Ac-Orn	NA	NA	NA	NA
ADMA	-6.17E-08	NA	NA	NA
alpha-AAA	2.16E-06	NA	NA	NA
c4 OH-Pro	NA	NA	NA	NA
Carnosine	NA	NA	NA	NA
Creatinine	NA	NA	NA	NA
DOPA	NA	NA	NA	NA
Dopamine	NA	NA	NA	NA
Histamine	NA	NA	NA	NA
Kynurenine	NA	-1.7701E-09	2.54E-07	3.0426E-07
Met-SO	NA	NA	NA	NA
Nitro-Tyr	NA	NA	NA	NA
PEA	NA	NA	NA	NA
Putrescine	4.20E-08	2.1738E-06	5.01E-06	3.5035E-06
SDMA	NA	NA	NA	NA
Serotonin	NA	NA	NA	NA
Spermidine	1.15E-06	1.8422E-05	1.77E-05	1.4483E-05

Spermine	3.26E-06	1.015E-05	6.19E-06	7.4952E-06
t4 OH-Pro	NA	NA	NA	NA
Taurine	NA	3.969E-06	1.67E-05	2.1748E-05
total DMA	NA	NA	NA	NA
lysoPC a C14:0	NA	NA	NA	NA
lysoPC a C16:0	3.55E-07	1.2622E-06	2.59E-07	1.723E-06
lysoPC a C16:1	1.02E-07	2.3818E-07	NA	2.4556E-07
lysoPC a C17:0	NA	NA	NA	NA
lysoPC a C18:0	6.51E-08	9.816E-07	6.46E-08	1.7348E-06
lysoPC a C18:1	3.43E-07	1.0055E-06	5.70E-07	1.2683E-06
lysoPC a C18:2	NA	3.5731E-07	NA	4.0174E-07
lysoPC a C20:3	NA	NA	NA	NA
lysoPC a C20:4	NA	1.6723E-07	NA	2.6681E-07
lysoPC a C24:0	NA	NA	NA	NA
lysoPC a C26:0	NA	NA	NA	NA
lysoPC a C26:1	NA	NA	NA	NA
lysoPC a C28:0	NA	NA	NA	NA
lysoPC a C28:1	NA	NA	NA	4.0231E-07
PC aa C24:0	NA	NA	NA	NA
PC aa C26:0	NA	NA	NA	NA
PC aa C28:1	2.68E-07	5.8548E-07	5.22E-07	7.5177E-07
PC aa C30:0	4.54E-06	1.1425E-05	6.50E-06	8.6767E-06
PC aa C30:2	9.00E-08	3.6153E-07	6.95E-08	3.1113E-07
PC aa C32:0	4.62E-06	2.0583E-05	6.88E-06	2.5513E-05
PC aa C32:1	2.23E-05	5.0744E-05	1.74E-05	3.1084E-05
PC aa C32:2	3.62E-06	8.4214E-06	1.76E-06	6.6577E-06
PC aa C32:3	1.38E-07	7.9097E-07	4.01E-08	9.6168E-07
PC aa C34:1	4.02E-05	0.00014211	6.25E-05	9.2514E-05
PC aa C34:2	2.19E-05	6.0397E-05	2.00E-05	4.7037E-05
PC aa C34:3	2.58E-06	7.4306E-06	5.61E-07	9.8642E-06
PC aa C34:4	2.83E-07	9.5227E-07	3.82E-08	1.6028E-06
PC aa C36:0	1.34E-07	1.2959E-06	NA	7.4125E-07
PC aa C36:1	4.52E-06	3.4957E-05	7.65E-06	2.3515E-05
PC aa C36:2	2.77E-05	5.8551E-05	5.20E-05	4.7741E-05
PC aa C36:3	1.12E-05	3.0188E-05	3.32E-06	3.1087E-05
PC aa C36:4	3.05E-06	1.3445E-05	3.41E-07	1.292E-05
PC aa C36:5	5.60E-07	4.2761E-06	9.33E-08	3.0967E-06
PC aa C36:6	1.60E-07	6.1133E-07	NA	4.5494E-07
PC aa C38:0	NA	8.4849E-07	NA	NA
PC aa C38:1	NA	6.3222E-07	NA	4.2675E-07
PC aa C38:3	3.15E-06	6.7562E-06	1.15E-06	7.5338E-06
PC aa C38:4	4.26E-06	9.5903E-06	2.22E-07	1.5427E-05
PC aa C38:5	1.65E-06	7.9235E-06	2.47E-07	1.2127E-05

PC aa C38:6	1.19E-06	7.018E-06	1.28E-07	5.393E-06
PC aa C40:1	NA	NA	NA	NA
PC aa C40:2	1.36E-07	3.0621E-07	4.53E-07	2.5278E-07
PC aa C40:3	2.11E-07	3.9493E-07	2.56E-07	5.6672E-07
PC aa C40:4	3.07E-07	7.1906E-07	3.67E-08	1.7644E-06
PC aa C40:5	3.37E-07	2.3798E-06	NA	4.5996E-06
PC aa C40:6	NA	4.5467E-06	NA	5.4424E-06
PC aa C42:0	NA	NA	NA	NA
PC aa C42:1	1.13E-08	7.4739E-08	4.18E-08	8.9866E-08
PC aa C42:2	3.83E-08	1.5089E-07	2.24E-07	1.8068E-07
PC aa C42:4	3.81E-08	8.4678E-08	6.70E-09	2.9814E-07
PC aa C42:5	NA	2.7867E-07	NA	6.4138E-07
PC aa C42:6	NA	NA	NA	NA
PC ae C30:0	2.43E-07	6.9646E-07	6.59E-07	4.7534E-07
PC ae C30:1	3.10E-08	3.9822E-07	1.18E-07	1.44E-07
PC ae C30:2	NA	NA	NA	NA
PC ae C32:1	8.53E-07	4.7356E-06	1.63E-06	2.2997E-06
PC ae C32:2	4.93E-08	1.2285E-06	1.63E-07	4.7448E-07
PC ae C34:0	1.07E-06	3.1081E-06	3.24E-07	3.6084E-06
PC ae C34:1	3.06E-06	1.4869E-05	4.66E-06	1.0962E-05
PC ae C34:2	5.83E-07	4.9741E-06	1.02E-06	3.0296E-06
PC ae C34:3	5.80E-08	1.4518E-06	1.21E-07	5.6325E-07
PC ae C36:0	1.62E-07	6.3797E-07	NA	7.7065E-07
PC ae C36:1	1.62E-06	7.8671E-06	7.24E-07	5.7614E-06
PC ae C36:2	1.68E-06	8.3147E-06	1.76E-06	6.2699E-06
PC ae C36:3	4.44E-07	2.9111E-06	3.84E-07	2.0121E-06
PC ae C36:4	7.37E-08	1.3983E-06	5.76E-08	9.7131E-07
PC ae C36:5	3.23E-08	1.755E-06	5.06E-08	7.6204E-07
PC ae C38:0	1.59E-07	8.4676E-07	7.84E-08	8.7035E-07
PC ae C38:1	1.45E-07	5.3626E-07	1.46E-07	5.3631E-07
PC ae C38:2	4.38E-07	1.6857E-06	3.11E-07	1.0908E-06
PC ae C38:3	3.78E-07	1.3455E-06	1.09E-07	1.107E-06
PC ae C38:4	2.61E-07	1.4219E-06	4.43E-08	1.4467E-06
PC ae C38:5	7.01E-08	2.1256E-06	6.16E-08	1.8633E-06
PC ae C38:6	4.05E-08	1.5034E-06	NA	8.9385E-07
PC ae C40:1	8.46E-08	6.2772E-07	NA	6.234E-07
PC ae C40:2	1.11E-07	3.5113E-07	7.22E-08	3.3143E-07
PC ae C40:3	6.75E-08	2.6041E-07	NA	3.0269E-07
PC ae C40:4	NA	3.6966E-07	NA	6.468E-07
PC ae C40:5	7.30E-08	6.6254E-07	3.43E-08	9.4321E-07
PC ae C40:6	5.86E-08	9.815E-07	NA	8.4158E-07
PC ae C42:0	NA	NA	NA	NA
PC ae C42:1	5.45E-08	2.2732E-07	9.86E-09	4.4842E-07

PC ae C42:2	4.73E-08	1.9016E-07	2.35E-08	3.4509E-07
PC ae C42:3	2.35E-08	1.8226E-07	NA	1.9601E-07
PC ae C42:4	NA	NA	NA	NA
PC ae C42:5	NA	NA	NA	NA
PC ae C44:3	5.83E-09	4.9497E-08	8.93E-09	1.6668E-07
PC ae C44:4	9.61E-09	8.6305E-08	-3.94E-11	1.3393E-07
PC ae C44:5	4.96E-09	7.8009E-08	-9.21E-09	1.1643E-07
PC ae C44:6	NA	NA	NA	NA
SM-OH C14:1	3.08E-07	1.8582E-06	1.06E-07	1.807E-06
SM-OH C16:1	2.04E-07	1.9457E-06	6.59E-08	2.6127E-06
SM-OH C22:1	2.55E-07	5.8137E-07	NA	5.7534E-07
SM-OH C22:2	2.49E-07	7.5497E-07	4.64E-08	8.5272E-07
SM-OH C24:1	5.70E-08	1.6936E-07	4.89E-08	1.2532E-07
SM C16:0	6.61E-06	3.5799E-05	1.62E-05	3.2544E-05
SM C16:1	2.54E-07	4.1846E-06	4.97E-07	3.4869E-06
SM C18:0	1.11E-06	3.9545E-06	1.04E-06	2.4702E-06
SM C18:1	1.56E-07	1.0599E-06	1.45E-07	5.9906E-07
SM C20:2	2.11E-08	8.9111E-08	NA	1.2558E-07
SM C22:3	NA	NA	NA	NA
SM C24:0	1.71E-06	4.4505E-06	3.56E-06	3.6189E-06
SM C24:1	2.53E-06	7.2192E-06	8.22E-06	9.3715E-06
SM C26:0	NA	NA	NA	NA
SM C26:1	2.64E-08	1.0841E-07	2.03E-07	1.2062E-07
H1	4.13E-06	NA	NA	NA

References

Salek, R. M., Steinbeck, C., Viant, M. R., Goodacre, R., & Dunn, W. B. (2013). The role of reporting standards for metabolite annotation and identification in metabolomic studies. *GigaScience, 2*, 13, doi:10.1186/2047-217X-2-13.
